# System analysis of the regulation of the immune response by CD147 and FOXC1 in cancer cell lines

**DOI:** 10.18632/oncotarget.24161

**Published:** 2018-01-11

**Authors:** Yu-Kui Shang, Can Li, Ze-Kun Liu, Ling-Min Kong, Ding Wei, Jing Xu, Zi-Ling Wang, Huijie Bian, Zhi-Nan Chen

**Affiliations:** ^1^ College of Life Sciences and Bioengineering, Beijing Jiaotong University, Beijing, 100044, China; ^2^ State Key Laboratory of Cancer Biology, Cell Engineering Research Center and Department of Cell Biology, Fourth Military Medical University, Xi'an, 71032, China

**Keywords:** CD147, immune response, FOXC1, gene expression, cancer cell line encyclopedia

## Abstract

CD147, encoded by *BSG*, is a highly glycosylated transmembrane protein that belongs to the immunological superfamily and expressed on the surface of many types of cancer cells. While CD147 is best known as a potent inducer of extracellular matrix metalloproteinases, it can also function as a key mediator of inflammatory and immune responses. To systematically elucidate the function of CD147 in cancer cells, we performed an analysis of genome-wide profiling across the Cancer Cell Line Encyclopedia (CCLE). We showed that CD147 mRNA expression was much higher than that of most other genes in cancer cell lines. CD147 varied widely across these cell lines, with the highest levels in the ovary (COLO704) and stomach (SNU668), intermediate levels in the lung (RERFLCKJ, NCIH596 and NCIH1651) and lowest levels in hematopoietic and lymphoid tissue (UT7, HEL9217, HEL and MHHCALL3) and the kidney (A704 and SLR20). Genome-wide analyses showed that CD147 expression was significantly negatively correlated with immune-related genes. Our findings implicated CD147 as a novel regulator of immune-related genes and suggest its important role as a master regulator of immune-related responses in cancer cell lines. We also found a high correlation between the expression of CD147 and FOXC1, and proved that CD147 was a direct transcriptional target of FOXC1. Our findings demonstrate that FOXC1 is a novel regulator of CD147 and confirms its role as a master regulator of the immune response.

## INTRODUCTION

CD147, a transmembrane glycoprotein, is expressed on all leukocytes, platelets and endothelial cells [1]. It has been implicated in various physiological and pathological activities through interacting with multiple partners, including cyclophilins, monocarboxylate transporters, caveolin-1 and integrins [2–4]. It can also function as a key mediator of inflammatory and immune response [4, 5]. Previous findings from functional experiments have shown that CD147 is an indicator of tumor prognosis [6] and it can promote cancer cell migration, invasion and metastasis by enhancing the activity of matrix metalloproteinases by digesting the components of the extracellular matrix in breast cancer, lymphoma, oral squamous cell carcinoma, glioma, melanoma, lung cancer, bladder and kidney carcinomas and ovarian cancer [7–13]. Because CD147 is important for the growth, survival and invasion of tumor cells, anti-CD147 reagents, such as anti-CD147 antibodies, peptide fragments of CD147 and siRNAs directed to CD147 are being explored as anti-tumor therapeutics [14–16]. It is speculated that CD147 interacting with many proteins representing various molecular or biological pathways contributes to malignant progression, eventually causing adverse clinical outcomes in cancer. Despite intense studies in the past, the exact essential role of CD147 in cancer cell remains unclear.

Cancer cell lines, originated from human tumors, have historically acted as the primary experimental model to investigate cancer biology and molecular pharmacology [17]. To study large-scale genomics, a suitable model is the cancer cell line collection of the Broad Institute and the Novartis Institutes for Biomedical Research, known as the Cancer Cell Line Encyclopedia (CCLE) panel [18]. The panel includes 1,036 cell lines from 24 different tissues of origin (*e.g.* breast, central nervous system, leukemia, colon, lung, melanoma, ovary, prostate and kidney). The CCLE is also one of the best characterized cell line collections for gene expression, chromosomal copy number and massively parallel sequencing data. The CCLE cell panel and associated databases provide a powerful approach to discover the biological features of novel genes and better understand the relationship of such genes with other functional genomic pathways.

In this study, we performed genome-wide expression analysis using the CCLE to shed light on the genomic determinants of CD147 expression. By analysis of this comprehensive CCLE cancer cell line dataset, we provided an unprecedented power not only for the comprehensive analysis of the roles of CD147 in cancer cells but also for the discovery of the trancriptional regulatory mechanism of CD147.

## RESULTS

### CD147 is a highly expressed gene in cancer cell lines

**Figure 1:**
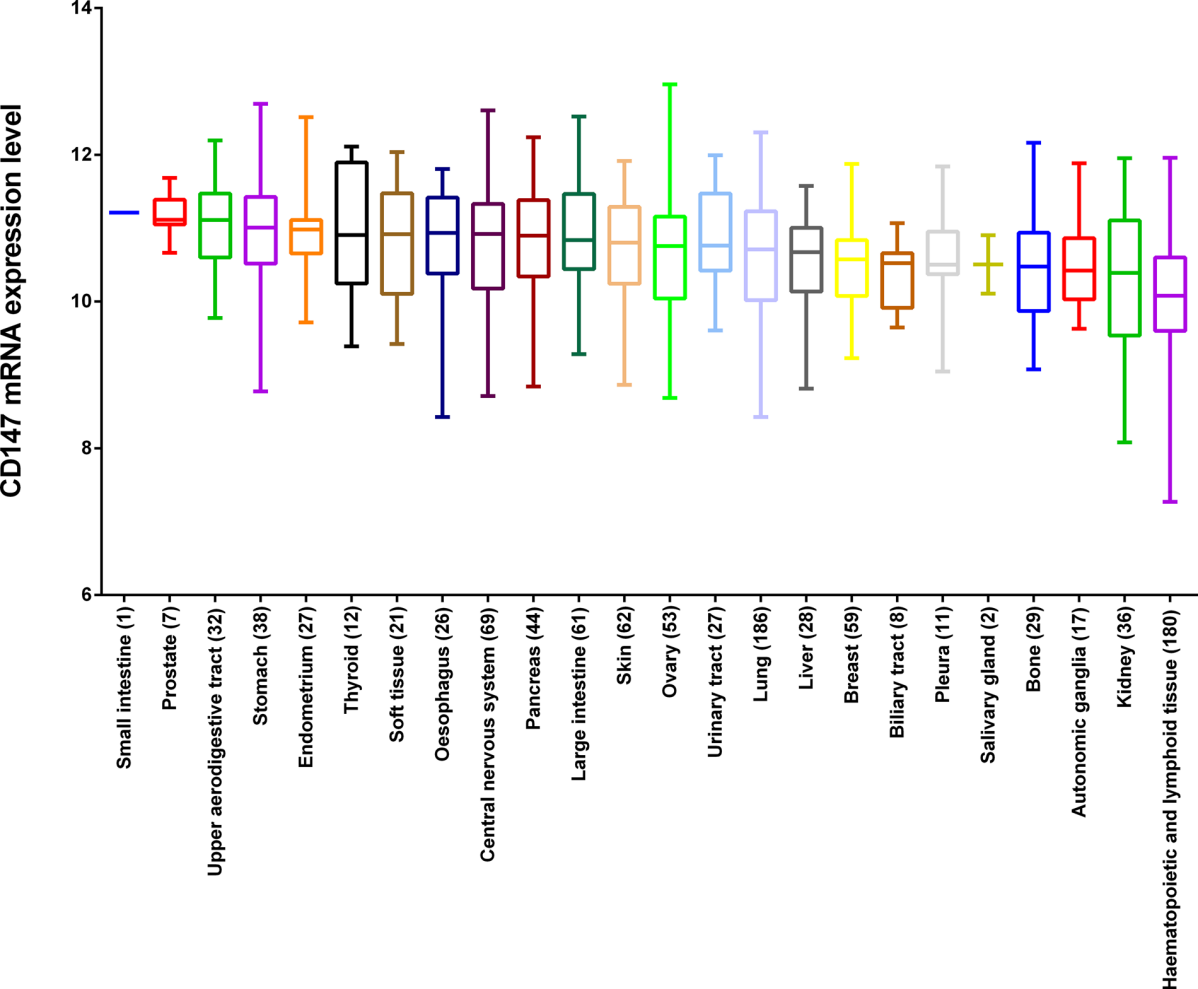
The mRNA expression profile of CD147 in the CCLE panel The expression values were obtained with Affymetrix U133+2 arrays. Quality filtering and normalization were performed using Robust Multi-array Average (RMA) and quantile normalization. The number in the brackets is the number of cell lines originated from the corresponding tissue.

**Figure 2:**
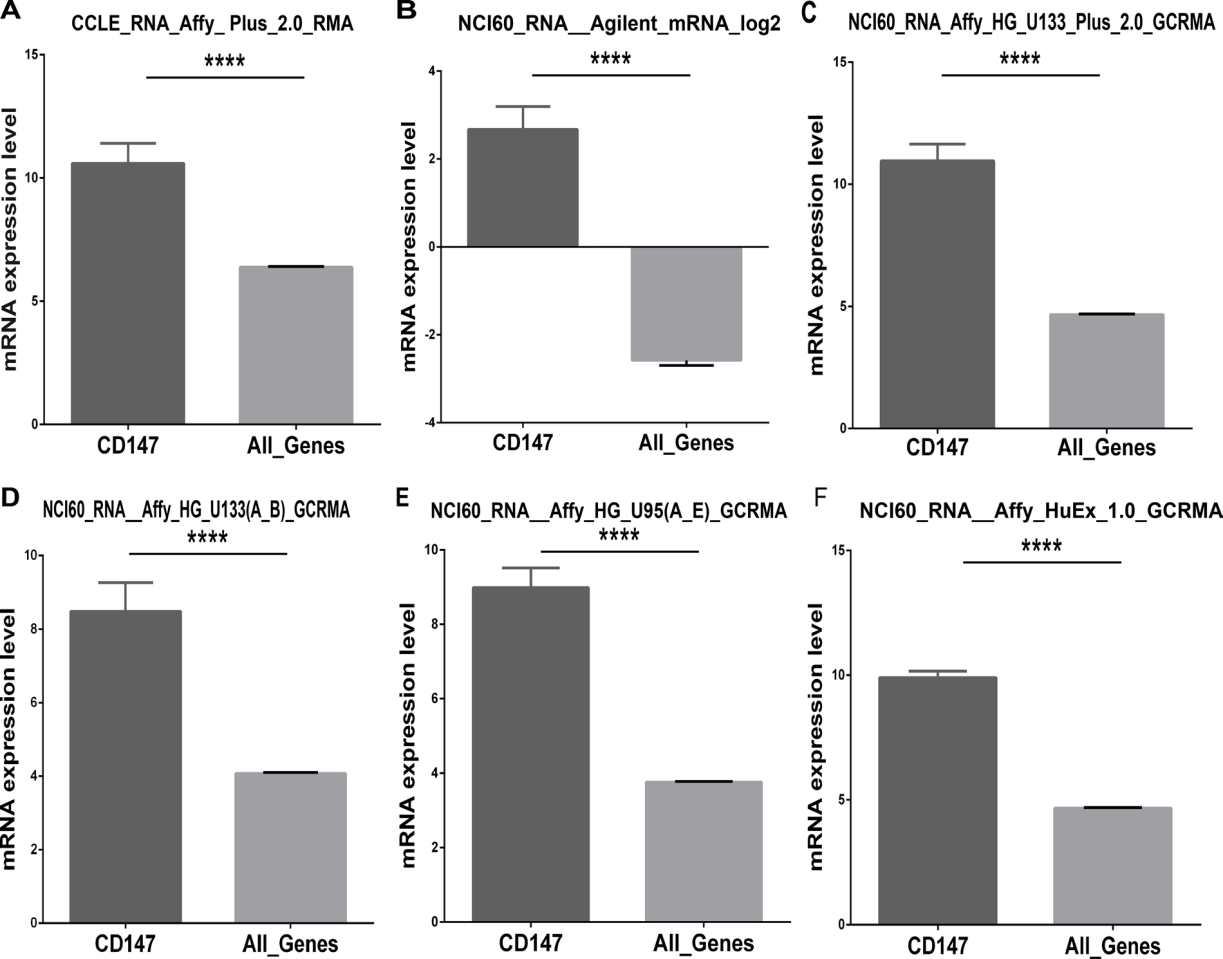
Comparison of the mRNA expression levels of CD147 and All_Genes in cancer cell lines from different microarray platforms (**A**) Expression values were obtained from CCLE. Five microarray platforms that have been exploited to generate transcriptome values in the NCI60: (**B**) Agilent WHG (Agilent Technologies; containing 41,000 probes), (**C**) Human Genome U133 Plus 2.0 (HG-U133 Plus 2.0; approximately 47,000 features), (**D**) Human Genome U133 (HG-U133a and b; approximately 44,000 features), (**E**) Affymetrix Human Genome U95 (HG-U95; approximately 60,000 features; Affymetrix Inc.) and (**F**) Affymetrix GeneChip Human Exon 1.0 ST (GH Exon 1.0 ST; approximately 850,000 features). The GC robust multi-array average (GCRMA) was used to normalize HG-U133 and HG-U95 arrays, whereas RMA was exploited for HG-U133 Plus 2.0 and HuEx 1.0 normalization. ^****^P < 0.0001, as assessed by Student’s *t*-test.

Recent studies have revealed that CD147 is a highly expressed gene in several malignant tumors, while the existing evidence lacks statistical power to draw a convincing conclusion. The larger number of cancer cell lines in the CCLE provides more clues about the expression of CD147 on many more cancer subtypes of different tissues of origin. In this study, we analyzed CD147 expression in the CCLE panel of cancer cell lines by microarray using Affymetrix U133+2 arrays, and revealed that CD147 was widely expressed in different types of cancer cell lines (Figure 1). Additionally, CD147 was more highly expressed in cancer cell lines than most of the other genes (Figure 2A). The expression level of CD147 in all 1,036 cancer cell lines was above the 95th percentile of gene expression across all genes found in the CCLE (All_Genes), indicating that all the cell lines highly expressed CD147 at a 1.78-fold variable level of difference between the lowest and highest CD147-expressing cell lines. Notably, cancer cell lines of central nervous system (SNU489, SF295 and DBTRG05MG) and digestive system origin (SNU668 and T84) showed relatively high CD147 expression. Whereas cancer cell lines of hematological origin (UT7, HEL9217, HEL, MHHCALL3, KASUMI2, BL70 and MOLT13) and kidney lines (A704 and SLR20) were among the lowest expressers of CD147. The reproducibility of CD147 mRNA expression measures was evaluated by comparing its transcriptional profile from the CCLE with the data from five different microarray platforms (Affymetrix HG-U95, HG-U133 a-b, HG-U133 Plus 2.0, Agilent WHG chips and Human Exon 1.0 ST) exploited in previous gene-expression studies of NCI60 (http://discover.nci.nih.gov/cellminer/) [19, 20], the cancer cell line collection of the National Cancer Institute Developmental Therapeutics Program (NCI-DTP). The results were highly concordant across the five platforms from NCI60, demonstrating the high reproducibility and accuracy of CD147 as a highly expressed gene in cancer cell lines (Figure 2B–2F). We further performed a pan-cancer analysis of data from Project Cognoma (https://github.com/cognoma/cognoma). The data provides the baseline gene expression profile of 20,469 unique genes based on RNA-seq for 7,036 cancer tissues including 28 tissue types and 33 cancer types from TCGA database (https://cancergenome.nih.gov/). Our results showed that CD147 was widely expressed in different types of cancer tissues (Supplementary Figure 1A). The expression level of CD147 in all 7,036 cancer samples was above the 95th percentile of gene expression across all genes found in the CCLE (Supplementary Figure 1B).

### CD147 is coexpressed with genes involved in immune response-related pathways and biology processes in cancer cell lines

Coexpression analysis has emerged as a powerful technique for single gene or multigene analysis of large-scale datasets. The underlying concept of gene coexpression analysis is ‘guilt-by-association’, in that groups of genes (known as coexpression) found to maintain a consistent expression relationship independent of phenotype are coregulated and may share a common biological role [21, 22]. Pearson’s correlation coefficient was calculated to assess the expression correlations between CD147 and all the other genes in the CCLE. In total, 1,339 genes were coexpressed with CD147 at a false discovery rate (FDR) < 0.01% and an absolute value correlation coefficient > 0.2. For 1,339 genes that were coexpressed with CD147, a notable bias was not found for positive correlations or negatively correlations (637 positively correlating versus 702 negatively correlating genes). To explore the potential effects of genes that are coexpressed with CD147 implicated in the molecular mechanism of CD147 in cancer cell lines, we further analyzed the functional Kyoto Encyclopedia of Genes and Genomes (KEGG) pathways [23] and Gene Ontology (GO) [24, 25] biology processes enriched with genes coexpressed with CD147 using Enrichr [26, 27]. The KEGG pathways and GO biology processes were then ranked based on the over-representation, and the significantly over-represented pathways and GO biology processes were shown in Table 1 and Table 2 (adjusted *P*-value < 0.1). Our results indicated that the genes coexpressed with CD147 were significantly enriched in 15 canonical pathways and 43 GO biology processes. Among these KEGG pathways, immunity-related pathways (hsa04662, hsa04666, hsa04650, hsa05340 and hsa04670) were significantly affected by genes that were coexpressed with CD147. For example, 20 of 73 total genes belonging to the B-cell receptor signaling pathway (hsa04662) were among the genes with a high expression correlation with CD147 (adjusted *P*-value = 0.0002). Sixteen genes belonging to the Fc gamma R-mediated phagocytosis pathway (hsa04666; adjusted *P*-value = 0.0486) were among the genes with a high expression correlation with CD147 out of 93 total genes. Many GO biology processes involving the immune response were also found among the over-represented terms. These included immune response-activating signal transduction (GO:0002757), antigen receptor-mediated signaling pathway (GO:0050851), B-cell receptor signaling pathway (GO:0050853), activation of the immune response (GO:0002253), immune response-activating cell surface receptor signaling pathway (GO:0002429), leukocyte activation (GO:0045321), immune response-regulating cell surface receptor signaling pathway (GO:0002768), regulation of leukocyte activation (GO:0002694), regulation of lymphocyte activation (GO:0051249), T cell activation (GO:0042110), regulation of immune effector process (GO:0002697), regulation of T cell activation (GO:0050863), T cell receptor signaling pathway (GO:0050852) and regulation of B cell activation (GO:0050864). In fact, all of the top eight most over-represented GO biology processes were directly related to the immune response. Within the group of genes that correlated with CD147, many encoded known components of the immune response, suggesting that CD147 plays important roles in regulating genes that are essential for the immune response.

**Table 1:** The KEGG pathways enriched with genes that coexpress with CD147 in cancer cell lines

Term	Overlap	*P*-value	Adjusted *P*-value
B cell receptor signaling pathway_Homo sapiens_hsa04662	20/73	8.10777E-07	0.000211613
Bacterial invasion of epithelial cells_Homo sapiens_hsa05100	19/78	6.64501E-06	0.000867174
Regulation of actin cytoskeleton_Homo sapiens_hsa04810	32/214	5.44627E-05	0.004738256
Protein processing in endoplasmic reticulum_Homo sapiens_hsa04141	26/169	0.000181939	0.011379825
Lysosome_Homo sapiens_hsa04142	21/123	0.000218004	0.011379825
Fc gamma R-mediated phagocytosis_Homo sapiens_hsa04666	16/93	0.001118042	0.048634835
Natural killer cell mediated cytotoxicity_Homo sapiens_hsa04650	20/135	0.001494003	0.055704956
Focal adhesion_Homo sapiens_hsa04510	25/202	0.003855139	0.085355505
Adherens junction_Homo sapiens_hsa04520	13/74	0.002710049	0.078591429
Primary immunodeficiency_Homo sapiens_hsa05340	9/37	0.001851544	0.060406616
Vibrio cholera infection_Homo sapiens_hsa05110	10/51	0.00415087	0.085355505
Inositol phosphate metabolism_Homo sapiens_hsa00562	12/71	0.005120678	0.095464067
Leukocyte transendothelial migration_Homo sapiens_hsa04670	17/118	0.004251424	0.085355505
N-Glycan biosynthesis_Homo sapiens_hsa00510	10/49	0.003233532	0.084395182
Carbohydrate digestion and absorption_Homo sapiens_hsa04973	9/45	0.005738694	0.099853278

**Table 2:** The GO biology processes enriched with genes that coexpress with CD147 in cancer cell lines

Term	Overlap	*P*-value	Adjusted *P*-value
Immune response-activating signal transduction (GO:0002757)	67/440	1.46271E-09	3.29E-06
Antigen receptor-mediated signaling pathway (GO:0050851)	32/127	1.6709E-09	3.29E-06
B cell receptor signaling pathway (GO:0050853)	17/33	2.79299E-09	3.66626E-06
Activation of immune response (GO:0002253)	69/487	1.1604E-08	1.14241E-05
Lymphocyte activation (GO:0046649)	49/304	5.29543E-08	4.17068E-05
Immune response-activating cell surface receptor signaling pathway (GO:0002429)	50/324	1.28934E-07	7.25344E-05
Leukocyte activation (GO:0045321)	55/373	1.16327E-07	7.25344E-05
Immune response-regulating cell surface receptor signaling pathway (GO:0002768)	57/444	3.74103E-06	0.00184152
Regulation of cell activation (GO:0050865)	54/420	6.48036E-06	0.002751334
Regulation of leukocyte activation (GO:0002694)	51/390	7.68529E-06	0.002751334
Negative regulation of ERBB signaling pathway (GO:1901185)	14/44	7.23179E-06	0.002751334
Regulation of ERBB signaling pathway (GO:1901184)	18/74	9.23422E-06	0.003030365
Regulation of lymphocyte activation (GO:0051249)	46/344	1.28679E-05	0.003897983
Regulation of defense response to virus by virus (GO:0050690)	11/29	1.83325E-05	0.00508506
Regulation of epidermal growth factor receptor signaling pathway (GO:0042058)	17/71	1.93692E-05	0.00508506
Negative regulation of epidermal growth factor receptor signaling pathway (GO:0042059)	13/43	2.40189E-05	0.005665519
T cell activation (GO:0042110)	31/198	2.44575E-05	0.005665519
Regulation of response to biotic stimulus (GO:0002831)	21/107	2.95489E-05	0.006464636
Regulation of defense response to virus (GO:0050688)	17/77	4.71126E-05	0.009764703
Hemostasis (GO:0007599)	55/478	8.70972E-05	0.017149432
Coagulation (GO:0050817)	54/472	0.00011518	0.020617238
Blood coagulation (GO:0007596)	54/472	0.00011518	0.020617238
Small gtpase mediated signal transduction (GO:0007264)	51/439	0.000126138	0.021597006
Regulation of immune effector process (GO:0002697)	35/264	0.000158429	0.025995632
Regulation of T cell activation (GO:0050863)	34/259	0.000230775	0.036132447
Phosphatidylinositol biosynthetic process (GO:0006661)	16/81	0.000239241	0.036132447
T cell receptor signaling pathway (GO:0050852)	18/99	0.000247734	0.036132447
Regulation of B cell activation (GO:0050864)	17/92	0.000308995	0.043457925
Activation of innate immune response (GO:0002218)	23/151	0.000373972	0.050521407
Toll-like receptor signaling pathway (GO:0002224)	20/122	0.000384876	0.050521407
Pattern recognition receptor signaling pathway (GO:0002221)	22/142	0.0004042	0.051346497
Platelet activation (GO:0030168)	28/205	0.00045525	0.056024237
Innate immune response-activating signal transduction (GO:0002758)	22/144	0.000478975	0.057157665
Phosphatidylinositol metabolic process (GO:0046488)	20/125	0.000507171	0.058742336
Regulation of B cell proliferation (GO:0030888)	12/54	0.000568696	0.06398637
Regulation of cell size (GO:0008361)	9/32	0.000667368	0.069160421
Maintenance of protein localization in organelle (GO:0072595)	8/25	0.00065458	0.069160421
Regulation of innate immune response (GO:0045088)	32/254	0.000659347	0.069160421
Positive regulation of innate immune response (GO:0045089)	26/190	0.000698128	0.070493046
T cell proliferation (GO:0042098)	10/41	0.000875861	0.086228484
Positive regulation of cell activation (GO:0050867)	33/272	0.000980389	0.091923174
Positive regulation of defense response (GO:0031349)	33/272	0.000980389	0.091923174
Positive regulation of leukocyte activation (GO:0002696)	32/262	0.001051807	0.096325984

### The immune response is negatively regulated by CD147 in cancer cell lines

**Figure 3:**
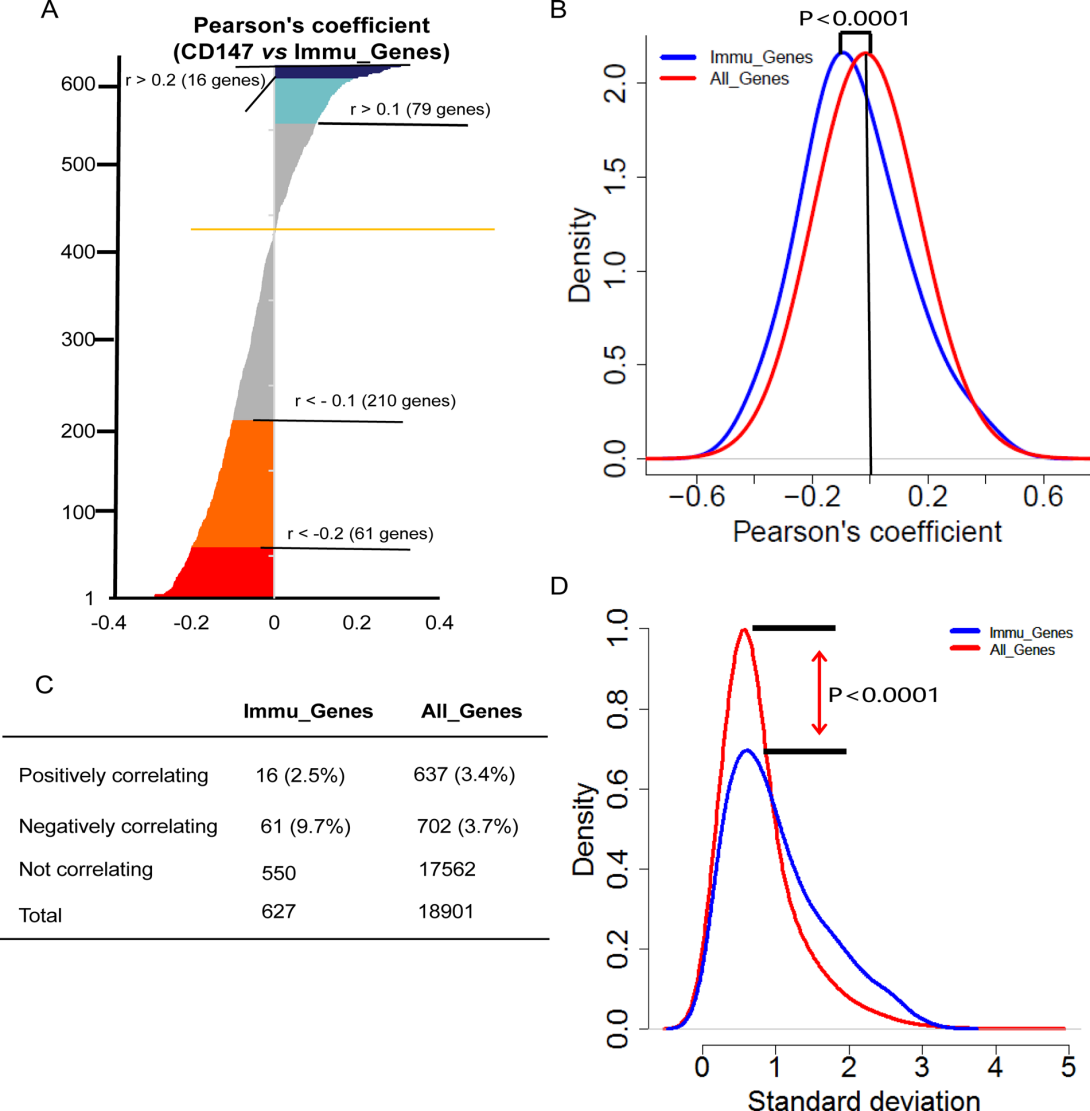
Coexpression of immu_genes with CD147 across the CCLE (**A**) Waterfall plot showing Pearson’s coefficients for the correlation between the CD147 transcript intensity in the CCLE and intensities of all Immu_Genes from InnateDB and IRIS databases. Pearson’s correlation coefficients were sorted from the highest positive (top) to lowest negative (bottom). The color codes are as follows: blue = Pearson’s r > 0.2 (26 genes, two-tailed *P* < 0.0001), turquoise = Pearson’s r > 0.1 (107 genes, two-tailed *P* < 0.0001), gray = non-significant correlations, orange = Pearson’s r < −0.1 (199 genes, two-tailed *P* < 0.0001) and red = Pearson’s r < −0.2 (57 genes, two-tailed *P* < 0.0001). (**B**) Density plot showing the distribution of Pearson’s correlations of Immu_Genes (blue) and All_Genes (red) with CD147. Immu_Genes showed a significant shift toward the left, indicating an excess of negative correlations with CD147 compared with the overall genome (*P* < 0.0001, two-tailed unpaired *t*-test). Y-axis: density-estimated values using a Gaussian kernel. (**C**) Contingency table showing numbers and percentages of Immu_Genes or all the genes that correlate positively or negatively or do not correlate with CD147. (**D**) Density plots showing the distribution of Immu_Genes (blue) and All_Genes (red) by standard deviation. The different shapes of the two distributions highlight the higher density of low standard deviations in the MNEG set (*P* < 0.0001, two-tailed unpaired *t*-test). X-axis: standard deviation of the gene expression across the CCLE. Y-axis: density values estimated using a Gaussian kernel model.

We compared the expression profile of CD147 with that of 662 immune-related genes (Immu_Genes) derived from the intersection of genes from InnateDB database (http://www.innatedb.com) [28] and Immunogenetic Related Information Source database (IRIS) (http://jtpc4.path.cam.ac.uk/immunedb/IRIS/home.htm) [29]. Next, we intersected the 662 genes with the list of genes in the CCLE. Finally, we obtained 627 Immu_Genes for further analysis. We found that the level of Immu_Genes mRNA expression was significantly lower than that of CD147 (P < 0.0001) and the average values of all the genes in the CCLE (*P* < 0.0001) as well (Supplementary Figure 2). The data in Figure 3A demonstrated a negative correlation of CD147 with 61 Immu_Genes. Of interest, a notably strong bias was found for negative correlations (16 positively correlating versus 61 negatively correlating genes, *P* < 0.0001) (Figure 3B). Moreover, the proportion of Immu_Genes that correlated negatively with CD147 was also higher than the positively correlating non-Immu_Genes (9.7%, 61 of 627 Immu_Genes, compared with 3.7%, 702 of 18,901 non-Immu_Genes; *P* < 0.001), suggesting that the Immu_Genes may be suppressed by CD147 in cancer cell lines, and CD147 may be a negative regulator for immune response processes (Figure 3C). To evaluate the reproducibility, we also compiled a list of 796 genes as Immu_Genes from 15 expert-curated KEGG immune-related pathways (http://immunet.princeton.edu./) [30] (Supplementary Figure 3A–3C). The results were highly concordant across the two lists of Immu_Genes from different sources, which independently validated the strong negative correlation between the immune response-related genes and CD147 in cancer cell lines, suggesting an immune response negatively regulates by CD147 in cancer cell lines. On the other hand, the Immu_Genes showed more variation than the other genes across the CCLE (Figure 3D), as assessed by comparing the standard deviations of the genes in these two sets (*P* < 0.0001), suggesting that all cancer cell lines may need a relatively unsteady expression of Immu_Genes.

By functional analysis based on GO of the genes correlated negatively with CD147, we found that most terms of GO biology processes were related to immune activation (Supplementary Table 7), such as lymphocyte activation (GO:0046649), leukocyte activation (GO:0045321), immune response-activating signal transduction, activation of the immune response (GO:0002253), B-cell activation (GO:0042113) and T-cell activation (GO:0042110). Together, these results demonstrate that CD147 plays a negative regulatory role in the immune response system in cancer cells.

**Figure 4:**
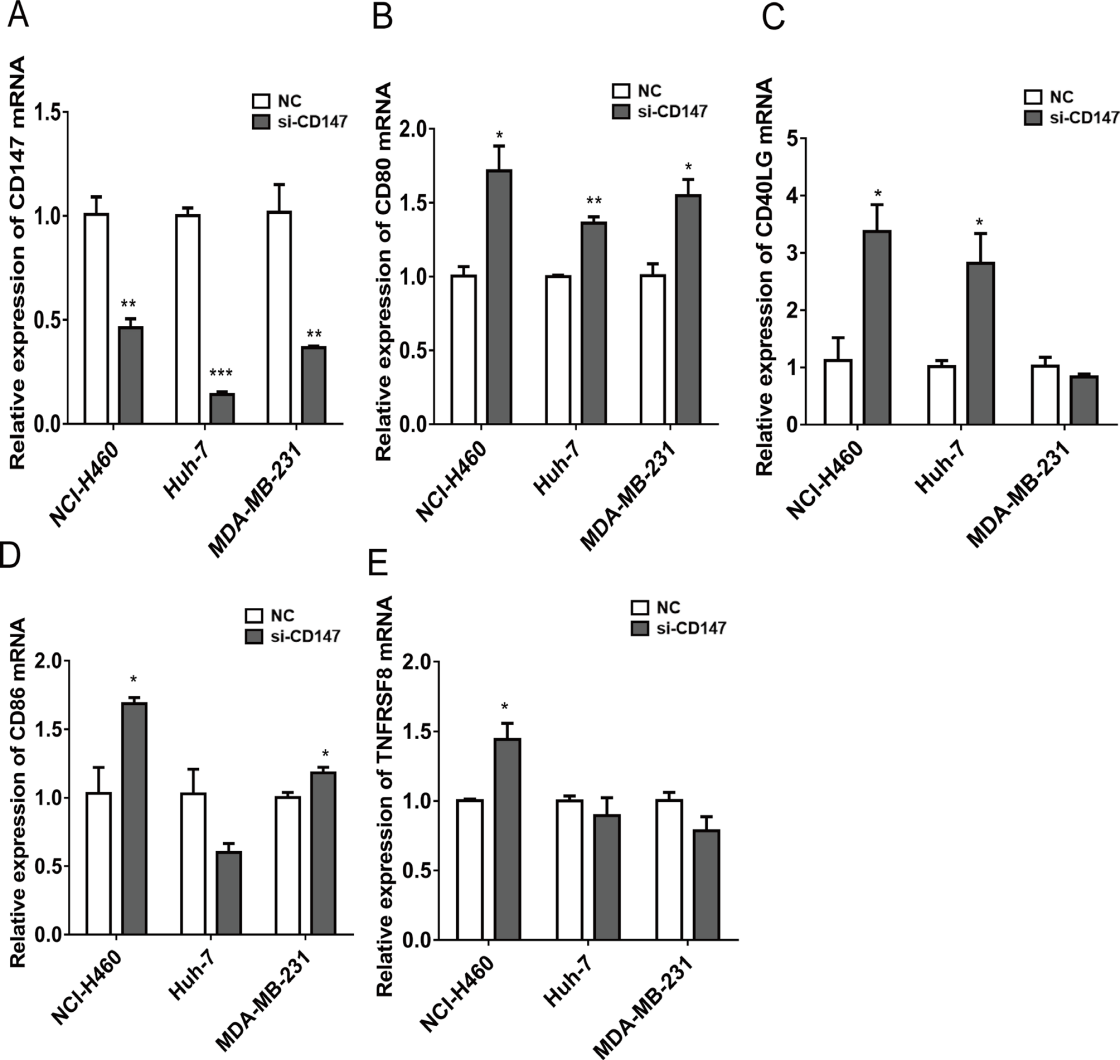
Quantitative RT-PCR showing mRNA expressions of immune-related genes (CD80, CD40LG, CD86 and TNFRSF8) in CD147-siRNA compared to control in different types of human tumor cell lines (NCI-H460, Huh-7 and MDA-MB-231) ^*^P < 0.05, ^**^P < 0.01.

To explain the immune response negatively regulated by CD147 *in vitro,* we used RNA interference (RNAi) for CD147 in three cancer cell lines (lung cancer NCI-H460, hepatocellular carcinoma Huh-7 and breast cancer MDA-MB-231) to test the mRNA expression of immune response-related genes. We focused on the immune response-related genes that negatively correlated with CD147 in CCLE and had been reported previously to express in cancer cells. Finally we selected CD80 [31, 32], CD40LG [33], CD86 [31] and TNFRSF8 [34, 35] for further study (Supplementary Table 1). Notably, silence of CD147, the mRNA expression of the four immune response-related genes in NCI-H460 cells, CD80 and CD40LG in Huh-7 cells, and CD80 and CD86 in MDA-MB-231 cells was increased (Figure 4A–4E).

### FOXC1 shows a significant correlation with CD147 and Immu_Genes

We next looked for a master transcription factor that regulates CD147 and Immu_Genes across the CCLE using Enrichr. Among the transcription factors which were enriched by Enrichr based on 1,339 genes that coexpressed with CD147, FOXC1 ranked the fifth most significantly over-represented transcription factor (adjusted *P*-value = 2.16E-14; Supplementary Table 2). While FOXC1 became the top one among the transcription factors which were enriched by Enrichr based on 702 genes that negatively correlated with CD147 (adjusted *P*-value = 1.22E-11; Supplementary Table 3). Additionally, FOXC1 was still significantly over-represented among the transcription factors enriched based on Immu_Genes from the intersection of InnateDB [28] and IRIS databases [29] (adjusted *P*-value = 4.50E-06; Supplementary Table 4). These results showed that most of the genes coexpressed with CD147 were involved in the immune response with a significant enrichment for genes that were under the transcriptional control of FOXC1.

**Figure 5:**
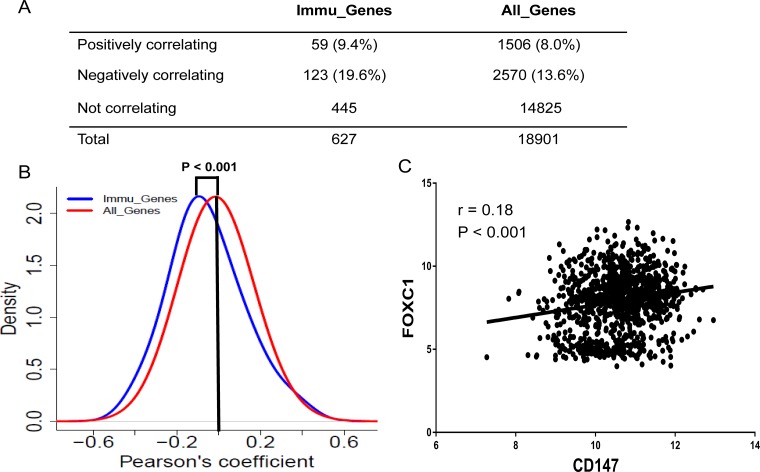
Coexpression of Immu_Genes with FOXC1 across the CCLE (**A**) Contingency table showing numbers and percentages of Immu_Genes or all the genes that correlate positively or negatively or do not correlate with FOXC1. (**B**) Density plot showing the distribution of Pearson’s correlations of Immu_Genes (blue) and all genes (red) with FOXC1. Immu_Genes show a significant shift toward the left, indicating an excess of negative correlations with FOXC1 compared with the overall genome (*P* < 0.0001, two-tailed unpaired *t*-test). Y-axis: density-estimated values using a Gaussian kernel. (**C**) Correlation between CD147 expression and FOXC1 expression in CCLE.

FOXC1 was also correlated negatively with 19.6% of the Immu_Genes; however, only 9.4% of the Immu_Genes were correlated positively with FOXC1 (FDR < 0.01% and the absolute value of the correlation coefficient > 0.2) (Figure 5A). Such negative correlation was greater in proportion than that of CD147 with Immu_Genes (19.6% versus 9.7%, *P* < 0.001) (Figure 5A and 5B, Figure 3C). Overall, FOXC1 and CD147 showed a significant correlation in their expression profiles across the CCLE (r = 0.18; *P* < 0.001) (Figure 5C). The expression level of FOXC1 was much higher than the mean expression level of all the genes and Immu_Genes in microarray from the CCLE (Supplementary Figure 4). Together, these results demonstrate that FOXC1 may be a key transcription factor that regulates CD147 and the immune response systems in cancer cells.

### Transcriptional regulation of CD147 is related to FOXC1

**Figure 6:**
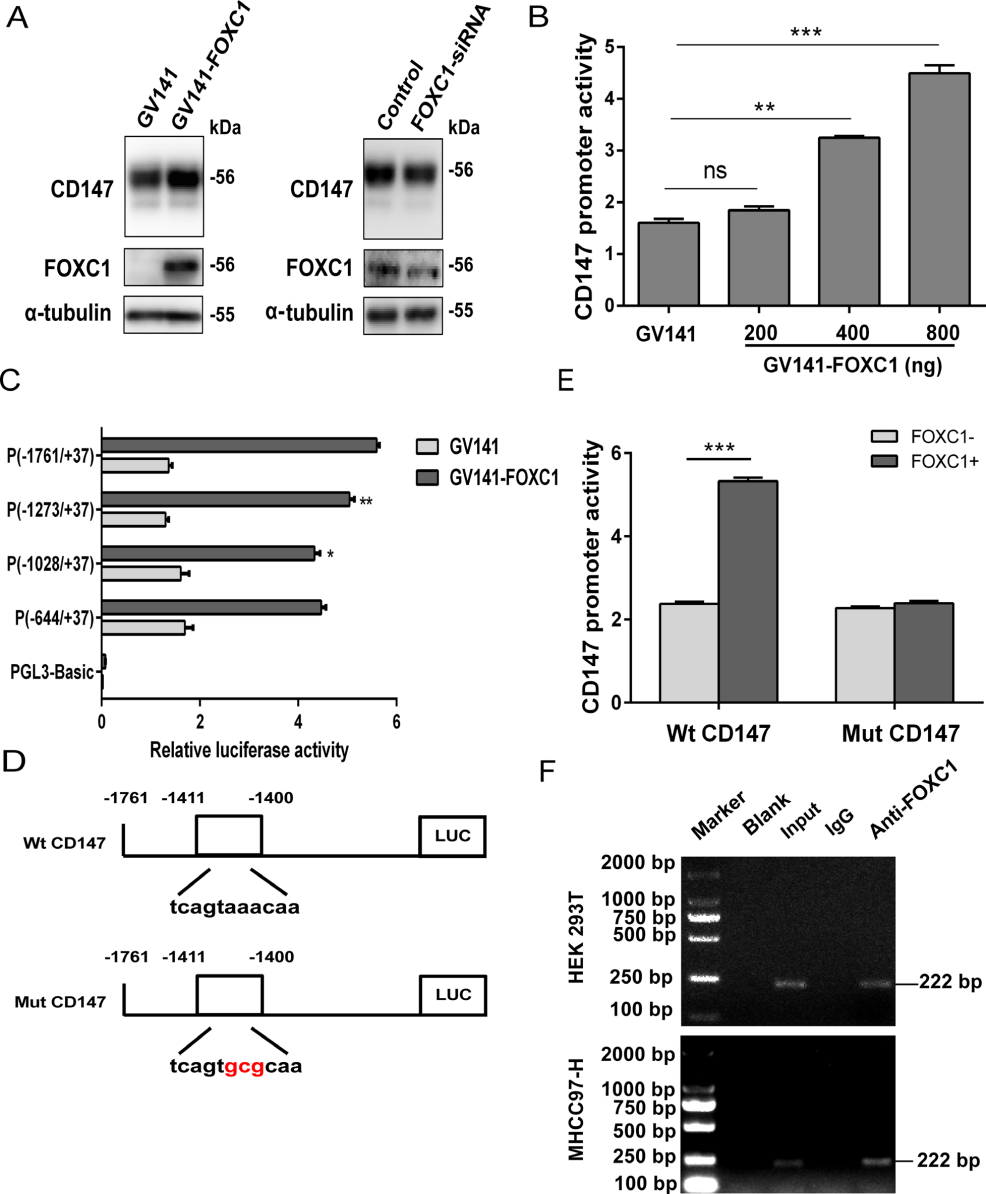
Transcriptional regulation of CD147 by FOXC1 (**A**) FOXC1 upregulated CD147 expression. HEK 293T cells were transfected with GV141-FOXC1 or FOXC1-siRNA, and the protein levels of CD147 in the transfected cells were detected using western blotting technique. (**B**) HEK 293T cells were cotransfected with the pGL3-Basic vector containing the human CD147 promoter (-1761/+37) and GV141-FOXC1, and the CD147 promoter activity was detected by the dual-luciferase reporter assay. (**C**) HEK 293T cells were cotransfected with different lengths of CD147 promoters and GV141-FOXC1, and the CD147 promoter activity was detected by the dual-luciferase reporter assay. (**D**) Schematic representation of the wild-type (Wt) and mutant (Mut) CD147 promoter regions. (**E**) HEK 293T cells were cotransfected with Wt CD147 or Mut CD147 promoters, and GV141-FOXC1, and the CD147 promoter activity was detected by the dual-luciferase reporter assay. (**F**) ChIP assay demonstrating the binding of FOXC1 to the CD147 promoter. Two percent of the lysate was used as the input control. ^**^P < 0.01, ^***^P < 0.001.

To further assess the relationship between FOXC1 and CD147, we tested whether CD147 expression could be modulated by FOXC1 expression in cells. Western blotting analyses showed that FOXC1 upregulated CD147 expression, whereas the knockdown of FOXC1 expression decreased CD147 expression in HEK 293T cells (Figure 6A). Using matrix-based nucleotide profiles of the transcription factor binding preference represented in the JASPAR database [36], we predicted the binding sites of FOXC1 in the human CD147 promoter region. Sites at positions −1411 to −1400 returned the highest scores of 10.761 (typical for moderate affinity binding). To further determine whether FOXC1 regulates CD147 transcription, a CD147 promoter luciferase construct, (-1761/+37) CD147, was cotransfected with GV141-FOXC1. A luciferase reporter assay showed that the cotransfection of GV141-FOXC1 and the CD147 promoter (-1761/+37) fragment into HEK 293T cells led to a FOXC1 dose-dependent increase in luciferase activity (Figure 6B). This result indicates that FOXC1 has an inducible effect on the activity of the CD147 promoter, which directly regulates the transcriptional expression of CD147.

Sequence analysis has revealed a perfect consensus FOXC1-binding site, 5′-GTAAACAA-3′, in the CD147 promoter [37]. Next, we used a series of deletion constructs to identify the minimal promoter required for CD147 activation. We found that the most critical region for the transcriptional activity of the CD147 promoter was located in position between −1273 and −1761, which involved this sequence (Figure 6C). We also observed that the promoter activity was partially abolished upon deletion of the sequence between −1028 and −1273, but the PCR-amplified fragment containing this region was not retrieved from the immunoprecipitates using an anti-FOXC1 antibody (data not shown). To further identify the CD147 promoter core region, we next generated a Mut CD147 construct that contained mutations in the putative FOXC1 binding elements by site-directed mutagenesis (Figure 6D). Site-directed mutagenesis showed that this FOXC1-binding site was critical for FOXC1-induced CD147 transactivation (Figure 6E). ChIP assay further confirmed FOXC1 binding directly to the CD147 promoter both in HEK 293T and MHCC97-H cell lines (Figure 6F). Altogether, these results provide molecular genetic evidences for the regulation of CD147 by FOXC1.

## DISCUSSION

By performing genome-wide cancer cell line analysis of gene expression microarrays in the extensively characterized CCLE panel, we demonstrated the highly coordinated expression of CD147 with the immune response-related genes that inhibit immune response-activating signal transduction, including inhibition of lymphocyte activation, leukocyte activation, B-cell activation and T-cell activation. We further demonstrated that FOXC1 is coexpressed with CD147 and Immu_Genes and showed that FOXC1 transcription influences the CD147 expression level. Our results provide the first evidence that CD147 is positively regulated by FOXC1, and extend prior reports indicating the relevance of CD147 and FOXC1 for the immune response system.

CD147, also known as extracellular matrix metalloproteinase inducer (EMMPRIN), is a member of the immunoglobulin family that is expressed on the surface of many types of tumor cells. CD147 has a high expression level in many malignant tumors, but its gene regulation and system biology have remained unknown. Here, we studied CD147 expression characteristics using the CCLE panel whose high-quality gene expression data enabled us to define a genomic signature for CD147 that was then used to identify the genome-wide relationships of CD147 with the other genes. Our data indicate that CD147 is overall highly expressed, although with a relatively stable expression level across the CCLE.

Genome-wide correlations across the CCLE demonstrated that CD147 is significantly coexpressed with a great proportion of immune response-related genes, especially those participating in immune response-activating signaling. Immu_Genes, especially those coexpressing with CD147 in cancer cells, highlight an intrinsic order in the regulation of the immune response. RNA interference (RNAi) for CD147 in cancer cells showed the role of the molecules in the tumor immune response negatively regulated by CD147. Specifically, these findings imply the importance of the inhibitory action of CD147 in the regulation of immune responses.

Using Enrichr tool, we found that FOXC1 is the one of most significantly over-represented transcription factors for genes that coexpress with CD147, especially for genes that are negatively correlated with CD147, leading us to analyze in further detail the relationship between CD147 and FOXC1. Accumulating evidences have showed that FOXC1 plays critical roles in cancer progression [38]. Elevated FOXC1 expression is associated with poor prognosis in various cancer types, in particular basal-like breast cancer (BLBC) [39–41]. Accordingly, our analysis demonstrated that the expression level of FOXC1 was much higher than the mean expression level of all the genes in cancer cell lines, and we provide molecular genetic evidence that FOXC1 regulates the transcription of CD147. Thus, the regulation of CD147 transcription by FOXC1 is consistent with the regulation of Immu_Genes by FOXC1. Our results suggest that FOXC1 may promote cancer progression by inhibiting the immune response system activation. Elucidation of the molecular basis of FOXC1 function would present itself as a potential therapeutic target for FOXC1-overexpressing tumors.

Our high throughput analysis approach is different from what was previously published data which using a limited number of cell lines in individual experiments. Here, we studied the convergence of the gene expression of a wide range of cancer cell lines, analyzed in their basal condition. The rationale for using cancer cell lines as an experimental model is that cancer cell lines retain the hallmarks of primary cancer cells [42, 43]. Compared with the cancer tissues that both tumors and normal are complex mixtures of cell types (cancer cells, infiltrating lymphocytes, stroma and blood vessels), the cancer cell lines are relatively homogeneous. Conversely, artifacts from the long-term culture of cell lines and their artificial *in vitro* culture conditions could affect our analysis [44].

In conclusion, our study demonstrated, under baseline conditions and without any system-altering methodology, the regulation of FOXC1 and CD147 in the immune response system in a panel of 1,036 cancer cell lines. Our study supports a critical role of CD147 regulated by FOXC1 in immune responses through suppression of immune response-activating signal transduction, and targeting CD147 could be a promising strategy for the treatment of cancer. To manipulate particular functions of CD147 for specific therapeutic targeting, more research is needed to better understand the mechanisms underlying CD147 in the regulation of immune responses under different biological and pathophysiological conditions.

## MATERIALS AND METHODS

### CCLE data

The CCLE project is an effort to conduct a detailed genetic characterization of a large panel of human cancer cell lines. The mRNA expression data of cancer cell lines were obtained through the CCLE′s online data portal site (https://portals.broadinstitute.org/CCLE/home). It provides the baseline gene expression profile of 18,901 unique genes (18,989 probes) for 1,036 human cancer cell lines collected from 37 tissue types and 24 cancer types. The gene expression profile data were obtained from Affymetrix U133+2 array platforms that have been exploited to generate transcriptome values. Raw Affymetrix CEL files were converted to a single value for each probe set using Robust Multi-array Average (RMA) and were normalized using quantile normalization [45].

### Functional enrichment analyses

The genes that coexpress with CD147 were used to carry out extensive analysis of functional categories GO biology processes terms and KEGG pathways and identify enrichment of transcription factors from the TRANSFAC [46] and JASPAR databases [36]. The analyses were conducted using the Enrichr tool (as of May 4th, 2016; http://amp.pharm.mssm.edu/Enrichr) [26, 27], which serves as an intuitive enrichment analysis web-based tool providing various types of visualization summaries of collective functions of gene lists. Statistical significance of the enrichment was assessed by *P*-value that has been adjusted using the Benjamini-Hochberg method for the correction for multiple hypotheses testing. Only the KEGG pathway, GO biology processes and transcription factors yielding an adjusted *P*-value < 0.05 were deemed to have enrichment of the genes that are coexpressed with CD147.

### Cell culture

The human embryonic kidney cell line HEK 293T, non-small-cell lung cancer cell line NCI-H460, human breast adenocarcinoma cell line MDA-MB-231, human hepatocellular carcinoma cell lines Huh-7 and MHCC97-H (all provided by Stem Cell Bank, Chinese Academy of Sciences) were cultured at 37°C in a humidified atmosphere containing 5% CO_2_ with Dulbecco′s Modified Eagle Medium (DMEM) (HyClone, UT, USA) containing 10% fetal bovine serum, 100 U/ml of penicillin and 100 mg/ml of streptomycin.

### Quantitative real-time PCR

Total RNA was extracted from NCI-H460, Huh-7 and MDA-MB-231 cells using the RNA Extraction Kit α (Omega Bio-tek, GA, USA) and then cDNA synthesis was performed using PrimeScriptTM RT Reagent Kit (TaKaRaBio, Otsu, Japan). Analysis of mRNA expression levels was performed in 96-well plates on Mx3005P connect Real-Time System (Agilent Technologies, CA, USA) with SYBR® Premix Ex TaqTM α (TaKaRaBio, Otsu, Japan) and specific primers. The primers were listed in Supplementary Table 5. The comparative Ct method was used to analyze gene expression differences between silencing CD147 expression (si-CD147) and control (NC) groups.

### RNA interference

Transfection of small interfering RNAs (siRNA) was performed using Lipofectamine 2000 (Invitrogen by Thermo Fisher Scientific, MA, USA). All siRNA sequences were synthesized by Shanghai GenePharma Co, Ltd. and were listed in Supplementary Table 6. The si-NC was used as a negative control under similar conditions.

### Dual-luciferase reporter assay

The 1,662-base pair cDNA containing the coding region of human FOXC1, named GV141-FOXC1, was purchased from Genechem (Shanghai, China). A reporter construct plasmid P(-1,761/+37) that contained a 1,798-bp genomic DNA fragment spanning the 5′ upstream region of CD147, as well as a series of deletion constructs, were generated by our laboratory [47]. The CD147 promoter plasmids containing the firefly luciferase reporter were cotransfected with GV141-FOXC1 and the internal control, pRL-TK (Promega, WI, USA) using Lipofectamine 2000 reagent (Invitrogen, CA, USA) and following the manufacturer′s protocol. Twenty-four hours after transfection, the cells were detected for luciferase activity using the Dual-Luciferase Reporter Assay System (Promega).

### Chromatin immunoprecipitation (ChIP)

ChIP was performed using the EZ ChIP™ Chromatin Immunoprecipitation Kit (Millipore, MA, USA) following the supplied protocol. Cell lysates were incubated with anti-FOXC1 antibody (PA1-807, Thermo Fisher Scientific, MA, USA) or IgG antibody (P2000-Y, Genia-Biotech, Beijing, China). The immunoprecipitated DNA was amplified using promoter-specific primers: forward 5′-TCA AAG GTT TGG CTC GTTCA-3′; reverse 5′-TAA CTG GAA AGG GGC AGG AAT-3′. The PCR products were analyzed on 2% agarose gels.

### Statistical analyses

Pearson product moment correlation was used to infer genes coexpressed with CD147 based on the data from the CCLE. Proportions of immune response-related genes (Immu_Genes) among CD147-correlated genes were compared with those among non-correlated ones using the Fisher test. Distribution plots for gene expression and gene correlations were generated by Gaussian kernel density estimation. The one-way analysis of variance (ANOVA) was used to compare the gene expression level between CD147 and other gene sets. All *P*-values were from two-tailed tests. All statistical analysis was performed using the R language (http://www.r-project.org/, version 3.3.0). Statistical significance was considered at a *P* value < 0.05.

## SUPPLEMENTARY MATERIALS FIGURES AND TABLES










