# Supplementary material for: System analysis of the regulation of the immune response by CD147 and FOXC1 in cancer cell lines
Source: Oncotarget. 2018 Jan 11;9(16):12918–31. doi: 10.18632/oncotarget.24161 (PMC5849184; doi:10.18632/oncotarget.24161)
Supplement: Supplementary file 1 [file oncotarget-09-12918-s001.pdf]

System analysis of the regulation of the immune response by CD147 and FOXC1 in cancer cell lines

SUPPLEMENTARY MATERIALS

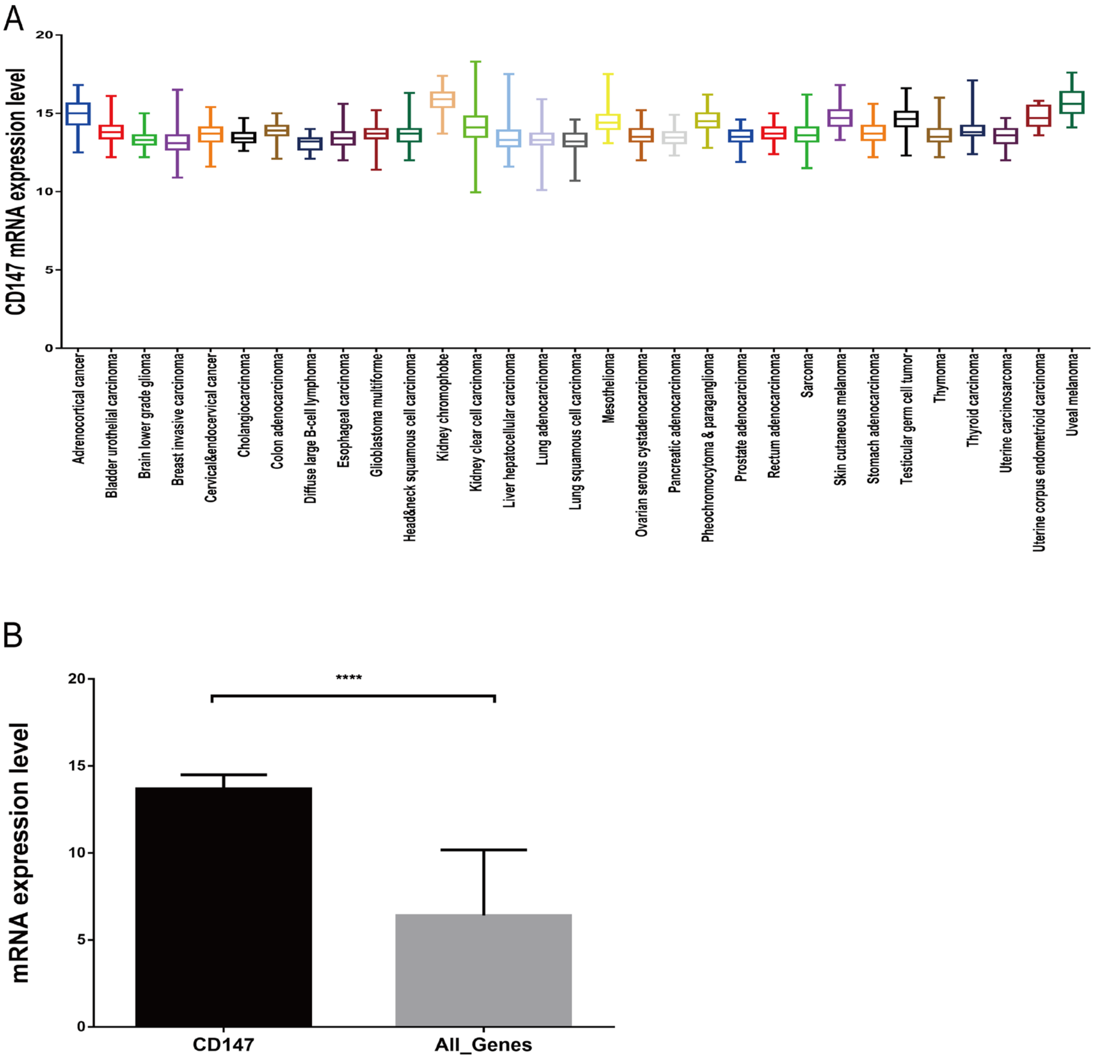

Supplementary Figure 1: CD147 is a highly expressed gene in cancer tissues from patients. (**A**) The mRNA expression profile of CD147 in the pan-cancer panel. (**B**) Comparison of the mRNA expression levels of CD147 and All_Genes in pan-cancer data. $^{****}P < 0.0001$, as assessed by Student's *t*-test.

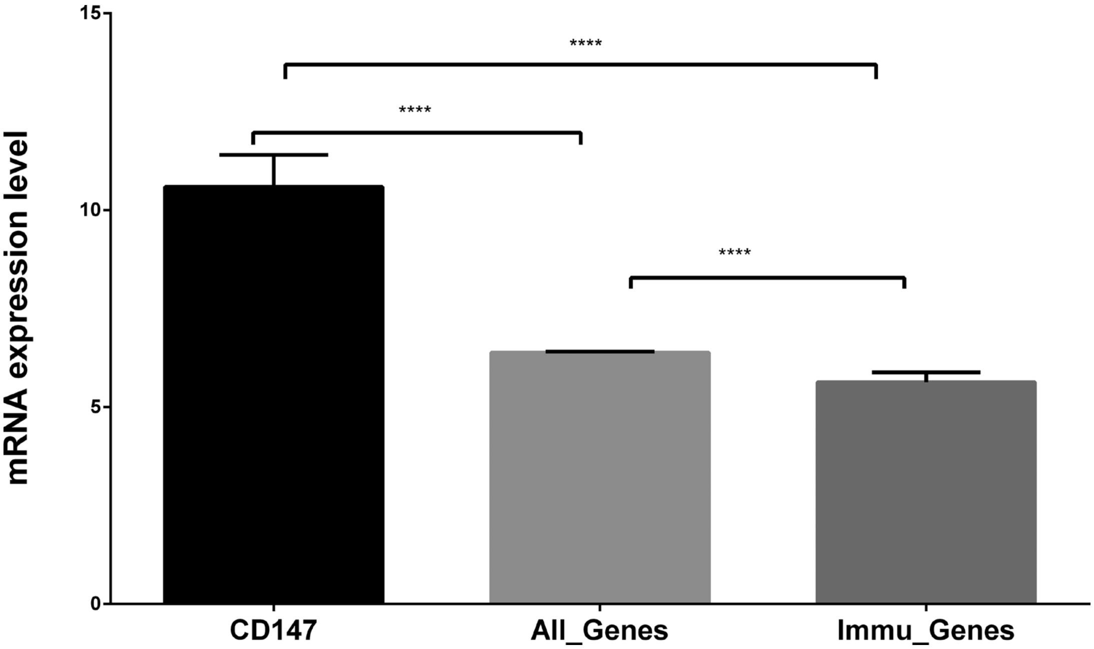

Supplementary Figure 2: The differential mRNA expression among CD147, All_genes and immu_genes in cancer cell lines from CCLE datasets. $^{****}P < 0.0001$.

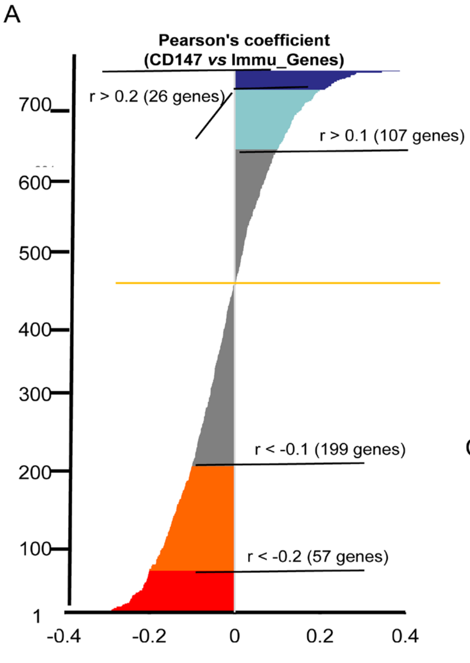

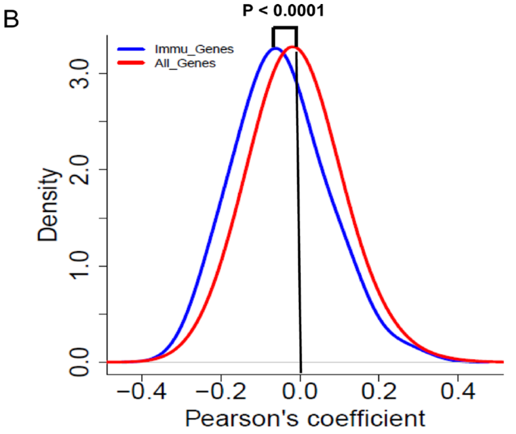

C

	Immu_Genes	All_Genes
Positively correlating	26 (3.5%)	637(3.4%)
Negatively correlating	57 (7.4%)	702 (3.7%)
Not correlating	653	17562
Total	736	18901

Supplementary Figure 3: Coexpression of immu_genes with CD147 across the CCLE. (**A**) Waterfall plot showing Pearson's coefficients for the correlation between the CD147 gene intensity in the CCLE and intensities of all Immu_Genes from 15 curated, immune-related KEGG pathways. Pearson's correlation coefficients were sorted from the highest positive (top) to lowest negative (bottom). The color codes are as follows: blue=Pearson's r > 0.2 (26 genes, two-tailed $P < 0.0001$), turquoise = Pearson's r > 0.1 (107 genes, two-tailed P < 0.0001), gray = non-significant correlations, orange = Pearson's r < -0.1 (199 genes, two-tailed $P < 0.0001$) and red = Pearson's r < -0.2 (57 genes, two-tailed $P < 0.0001$). (**B**) Density plot showing the distribution of Pearson's correlations of Immu_Genes (blue) and All_Genes (red) with CD147. Immu_Genes showed a significant shift toward the left, indicating an excess of negative correlations with CD147 compared with the overall genome ($P < 0.0001$, two-tailed unpaired t-test). Y-axis: density-estimated values using a Gaussian kernel. (**C**) Contingency table showing numbers and percents of Immu_Genes or all the genes that correlate positively or negatively or do not correlate with CD147.

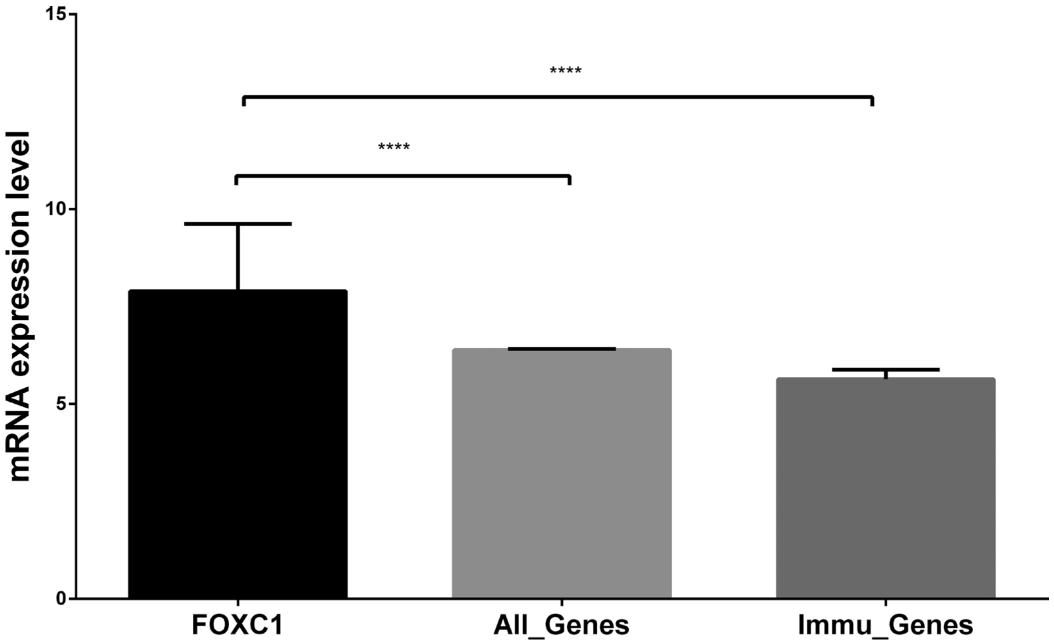

Supplementary Figure 4: The differential mRNA expression among FOXC1, All_genes and immu_genes in cancer cell lines from CCLE datasets. $^{****}P < 0.0001$.

Supplementary Table 1: Four immune response-related genes negatively correlated with CD147 in CCLE

Term	r	*P*	Adjusted *P*-value
CD80	-0.15	5.80E-07	3.35E-06
CD40LG	-0.15	8.83E-07	5.35E-06
CD86	-0.15	9.25E-07	5.57E-06
TNFRSF8	-0.14	1.18E-05	5.51E-05

Supplementary Table 2: The transcription factors enriched by Enrichr based on 1,339 genes that coexpressed with CD147. See_Supplementary_Table 2

Supplementary Table 3: The transcription factors enriched by Enrichr based on the 702 genes that negatively correlated with CD147. See_Supplementary_Table 3

Supplementary Table 4: The transcription factors enriched by Enrichr based on the Immu_Genes from InnateDB and IRIS databases. See_Supplementary_Table 4

Supplementary Table 5: Gene-specific primers for quantitative RT-PCR

Target gene	Forward primer (5' to 3')	Reverse primer (5' to 3')
CD147	ACTCCTCACCTGCTCCTTGA	GCCTCCATGTTCAGGTTCTC
CD80	AAACTCGCATCTACTGGCAAA	GGTTCTTGTACTCGGGCCATA
CD40LG	ACTTGGTAACCCTGGAAA	GGCTGGCTATAAATGGAG
CD86	CTCTGGTGCTGCTCCTCT	GGGTCCAACTGTCCGAAT
TNFRSF8	CAGGATATGGCTGAGAAGGAC	GACCACCACAACCAACACC
GAPDH	TGATGACATCAAGAAGGTGGTGAAG	TCCTTGGAGGCCATGTGGGCCAT

Supplementary Table 6: RNAi oligonucleotides for target gene silencing

Target gene	RNAi oligonucleotides (5' to 3')
si-CD147	GTACAAGATCACTGACTCT
si-NC	UUCUCCGAACGUGUCACGUTT

Supplementary Table 7: The GO biology processes enriched with genes that negatively correlate with CD147 in cancer cell lines. See_Supplementary_Table 7
