# Supplementary material for: System analysis of the regulation of the immune response by CD147 and FOXC1 in cancer cell lines
Source: Oncotarget. 2018 Jan 11;9(16):12918–31. doi: 10.18632/oncotarget.24161 (PMC5849184; doi:10.18632/oncotarget.24161)
Supplement: Supplementary file 2 [file oncotarget-09-12918-s002.docx]

**Supplementary Table 2: The transcription factors enriched by Enrichr based on 1,339 genes that coexpressed with CD147**

| **Term** | **Overlap** | **P-value** | **Adjusted P-value** |
| --- | --- | --- | --- |
| NFKB1 (human) | 281/3497 | 4.73755E-21 | 1.48285E-18 |
| WT1 (human) | 228/2689 | 1.97513E-19 | 3.09107E-17 |
| SREBF1 (human) | 218/2587 | 2.88381E-18 | 3.00877E-16 |
| RELA (human) | 222/2775 | 3.13259E-16 | 2.15516E-14 |
| FOXC1 (human) | 739/13142 | 3.44275E-16 | 2.15516E-14 |
| PITX1 (human) | 138/1448 | 2.21871E-15 | 7.71617E-14 |
| PITX2 (human) | 262/3528 | 1.80939E-15 | 7.71617E-14 |
| USF2 (human) | 277/3792 | 1.48226E-15 | 7.71617E-14 |
| E2F1 (human) | 299/4207 | 2.18404E-15 | 7.71617E-14 |
| TFAP2D (human) | 129/1324 | 4.43401E-15 | 1.38785E-13 |
| SP3 (human) | 128/1332 | 1.46066E-14 | 4.15625E-13 |
| RUNX1 (human) | 276/3857 | 1.83363E-14 | 4.78272E-13 |
| NR5A2 (human) | 273/3815 | 2.67783E-14 | 6.44739E-13 |
| GFI1 (human) | 133/1427 | 3.52952E-14 | 7.891E-13 |
| CRX (human) | 128/1362 | 6.48642E-14 | 1.3535E-12 |
| FOXF2 (human) | 128/1379 | 1.47128E-13 | 2.87819E-12 |
| PCBP1 (human) | 125/1360 | 5.47263E-13 | 1.00761E-11 |
| ZNF148 (human) | 139/1591 | 8.02516E-13 | 1.39549E-11 |
| TEAD4 (human) | 124/1354 | 8.58356E-13 | 1.41403E-11 |
| TFAP2A (human) | 254/3613 | 1.76342E-12 | 2.75975E-11 |
| E2F6 (human) | 122/1358 | 4.33246E-12 | 6.45744E-11 |
| PPARG (human) | 250/3580 | 5.40277E-12 | 7.68667E-11 |
| CACYBP (human) | 121/1350 | 6.14859E-12 | 8.36743E-11 |
| CEBPD (human) | 130/1495 | 6.44865E-12 | 8.41011E-11 |
| KLF5 (human) | 126/1445 | 1.18738E-11 | 1.4866E-10 |
| NR1H3 (human) | 122/1385 | 1.41938E-11 | 1.64543E-10 |
| NFIC (human) | 292/4403 | 1.4027E-11 | 1.64543E-10 |
| SP1 (mouse) | 180/2360 | 1.57377E-11 | 1.75924E-10 |
| IKZF1 (human) | 121/1373 | 1.68614E-11 | 1.81986E-10 |
| RARA (human) | 121/1379 | 2.18222E-11 | 2.27679E-10 |
| MIR133B (human) | 127/1500 | 5.85973E-11 | 5.91644E-10 |
| KLF4 (human) | 126/1485 | 6.18187E-11 | 6.04664E-10 |
| SP1 (human) | 121/1406 | 6.78462E-11 | 6.43511E-10 |
| TCFAP2A (human) | 118/1367 | 1.00534E-10 | 9.25504E-10 |
| ZBTB7A (human) | 118/1373 | 1.28772E-10 | 1.15159E-09 |
| MAX (human) | 151/1934 | 1.90757E-10 | 1.65853E-09 |
| RXRA (human) | 120/1422 | 2.50162E-10 | 2.06054E-09 |
| ZFHX3 (human) | 125/1504 | 2.48853E-10 | 2.06054E-09 |
| CREB1 (human) | 196/2749 | 3.8021E-10 | 3.05143E-09 |
| ATF4 (human) | 117/1398 | 6.7232E-10 | 5.26091E-09 |
| NR1I2 (human) | 116/1387 | 8.30152E-10 | 6.18661E-09 |
| HIF1A (human) | 128/1586 | 8.26196E-10 | 6.18661E-09 |
| TP63 (human) | 119/1443 | 1.06717E-09 | 7.76803E-09 |
| SRF (human) | 230/3420 | 1.2746E-09 | 9.06707E-09 |
| EGR1 (mouse) | 129/1617 | 1.3737E-09 | 9.55481E-09 |
| SMARCA2 (human) | 124/1536 | 1.53146E-09 | 1.04206E-08 |
| KLF11 (human) | 115/1388 | 1.62636E-09 | 1.08309E-08 |
| YY1 (human) | 363/6042 | 2.88319E-09 | 1.88008E-08 |
| NRF1 (mouse) | 131/1676 | 3.27586E-09 | 2.09254E-08 |
| SNAI1 (human) | 116/1428 | 3.90331E-09 | 2.34949E-08 |
| SNAI2 (human) | 116/1428 | 3.90331E-09 | 2.34949E-08 |
| TCF3 (human) | 116/1428 | 3.90331E-09 | 2.34949E-08 |
| NFE2 (human) | 187/2681 | 5.0979E-09 | 3.01065E-08 |
| MTF1 (human) | 116/1440 | 6.03545E-09 | 3.49832E-08 |
| THRB (human) | 150/2034 | 8.10312E-09 | 4.61141E-08 |
| KLF13 (human) | 116/1453 | 9.59498E-09 | 5.36291E-08 |
| GATA2 (human) | 549/9985 | 1.18276E-08 | 6.49482E-08 |
| RELB (human) | 116/1469 | 1.67738E-08 | 9.05208E-08 |
| HINFP (human) | 204/3047 | 1.91172E-08 | 1.01419E-07 |
| TP53 (human) | 194/2866 | 2.1432E-08 | 1.11804E-07 |
| GLI2 (human) | 115/1465 | 2.61394E-08 | 1.34125E-07 |
| JUN (human) | 199/2976 | 3.24024E-08 | 1.6358E-07 |
| ETS1 (human) | 294/4827 | 5.45913E-08 | 2.71223E-07 |
| ELF3 (human) | 115/1488 | 5.64541E-08 | 2.76096E-07 |
| NRF1 (human) | 107/1356 | 6.463E-08 | 3.11218E-07 |
| SMAD4 (mouse) | 120/1580 | 6.86488E-08 | 3.25562E-07 |
| ESR1 (human) | 116/1513 | 7.29521E-08 | 3.40806E-07 |
| IRF8 (human) | 118/1550 | 7.92412E-08 | 3.64742E-07 |
| LTF (human) | 107/1363 | 8.20979E-08 | 3.72415E-07 |
| MAPK14 (human) | 96/1190 | 1.24581E-07 | 5.57054E-07 |
| RELA (mouse) | 123/1656 | 1.43549E-07 | 6.32827E-07 |
| MIB2 (human) | 113/1490 | 1.8219E-07 | 7.9202E-07 |
| NFYA (human) | 171/2549 | 2.94598E-07 | 1.26314E-06 |
| REPIN1 (human) | 110/1466 | 4.34636E-07 | 1.83839E-06 |
| FOXJ1 (human) | 111/1484 | 4.43133E-07 | 1.84653E-06 |
| ZBTB16 (human) | 116/1572 | 4.5316E-07 | 1.84653E-06 |
| NFKB1 (mouse) | 209/3284 | 4.54259E-07 | 1.84653E-06 |
| YY1 (mouse) | 123/1700 | 5.18952E-07 | 2.08246E-06 |
| SREBF2 (human) | 102/1361 | 1.2248E-06 | 4.8527E-06 |
| TFAP2C (human) | 109/1485 | 1.29547E-06 | 5.06852E-06 |
| LEF1 (human) | 276/4658 | 1.52743E-06 | 5.90229E-06 |
| Myb (mouse) | 132/1902 | 1.55225E-06 | 5.92504E-06 |
| APEX1 (human) | 102/1373 | 1.76174E-06 | 6.64367E-06 |
| HNF4A (human) | 144/2127 | 1.80444E-06 | 6.7237E-06 |
| ATF2 (human) | 110/1517 | 1.96029E-06 | 7.21848E-06 |
| TEAD2 (human) | 108/1486 | 2.21472E-06 | 8.06057E-06 |
| NR1H2 (human) | 47/479 | 2.30873E-06 | 8.30613E-06 |
| MZF1_1-4 (human) | 174/2705 | 2.72779E-06 | 9.70224E-06 |
| SMAD4 (human) | 110/1542 | 3.92765E-06 | 1.3813E-05 |
| MEF2A (human) | 178/2800 | 4.04566E-06 | 1.40699E-05 |
| RUNX2 (human) | 105/1458 | 4.48172E-06 | 1.54152E-05 |
| MZF1 (human) | 101/1389 | 4.69379E-06 | 1.59691E-05 |
| MIR138 (human) | 99/1356 | 4.99625E-06 | 1.68153E-05 |
| STAT1 (human) | 116/1667 | 6.22386E-06 | 2.07241E-05 |
| USF1 (human) | 103/1441 | 7.46377E-06 | 2.45912E-05 |
| EGR1 (human) | 58/681 | 9.45208E-06 | 3.08177E-05 |
| GATA1 (human) | 169/2679 | 1.1095E-05 | 3.58015E-05 |
| MYC (human) | 94/1302 | 1.31365E-05 | 4.19565E-05 |
| NR3C1 (human) | 110/1590 | 1.38229E-05 | 4.37026E-05 |
| PRDM1 (human) | 103/1468 | 1.53548E-05 | 4.80606E-05 |
| MYOG (human) | 99/1404 | 1.87717E-05 | 5.81736E-05 |
| MYC (mouse) | 115/1699 | 2.15001E-05 | 6.59757E-05 |
| SP3 (mouse) | 83/1129 | 2.34113E-05 | 7.11432E-05 |
| NFIA (human) | 99/1416 | 2.56859E-05 | 7.73046E-05 |
| TCF4 (human) | 244/4210 | 3.21448E-05 | 9.58222E-05 |
| TBP (human) | 156/2486 | 3.24958E-05 | 9.59544E-05 |
| XBP1 (human) | 51/605 | 4.10532E-05 | 0.00012009 |
| FOXL1 (human) | 319/5755 | 4.34185E-05 | 0.000125833 |
| TFAP2A (mouse) | 111/1663 | 5.15325E-05 | 0.000147979 |
| SND1 (human) | 100/1473 | 6.7629E-05 | 0.000192435 |
| ETV4 (human) | 96/1403 | 7.23355E-05 | 0.000203973 |
| E2F1 (mouse) | 183/3056 | 7.61983E-05 | 0.000212947 |
| RBPJ (human) | 103/1535 | 7.99873E-05 | 0.000221558 |
| SAMD9L (human) | 96/1415 | 9.6672E-05 | 0.000265424 |
| PLAU (human) | 93/1362 | 0.000100472 | 0.000273459 |
| SPI1 (human) | 108/1638 | 0.00010218 | 0.000275711 |
| UBTF (human) | 100/1493 | 0.000108236 | 0.000289555 |
| GATA6 (human) | 97/1447 | 0.000133446 | 0.000353971 |
| IRF2 (human) | 95/1412 | 0.000138677 | 0.000361717 |
| ZNF354C (human) | 96/1430 | 0.00013765 | 0.000361717 |
| MYOD1 (mouse) | 109/1672 | 0.000142938 | 0.000369749 |
| CEBPB (human) | 95/1420 | 0.000166939 | 0.000428296 |
| NR5A1 (human) | 93/1389 | 0.000190398 | 0.000484508 |
| PGR (human) | 96/1447 | 0.000203004 | 0.000512422 |
| ZNF281 (human) | 70/986 | 0.00026072 | 0.000652842 |
| LEF1 (mouse) | 110/1730 | 0.000321462 | 0.000798552 |
| POU2F1 (human) | 92/1399 | 0.000360922 | 0.000889516 |
| RORB (human) | 72/1035 | 0.000369267 | 0.000902974 |
| ETS1 (mouse) | 106/1681 | 0.000542142 | 0.001315429 |
| NR2F1 (human) | 73/1073 | 0.000605752 | 0.001458465 |
| TP53 (mouse) | 83/1266 | 0.000753275 | 0.001799809 |
| HNF1A (human) | 114/1855 | 0.000808397 | 0.00191688 |
| CBEPA (human) | 88/1366 | 0.000872679 | 0.002053748 |
| FOXF1 (human) | 87/1352 | 0.000961723 | 0.002246414 |
| FOS (human) | 89/1394 | 0.001054248 | 0.002444294 |
| ELK4 (human) | 92/1453 | 0.001108359 | 0.002550856 |
| MYB (human) | 89/1409 | 0.001419461 | 0.003219502 |
| POU2F2 (human) | 160/2792 | 0.001413943 | 0.003219502 |
| ARNT (human) | 83/1303 | 0.00163184 | 0.003648328 |
| ELK1 (human) | 94/1511 | 0.001627439 | 0.003648328 |
| ELK1 (mouse) | 105/1727 | 0.001786651 | 0.003966112 |
| GABPA (human) | 13/108 | 0.002207504 | 0.004865836 |
| FOXA1 (mouse) | 103/1705 | 0.002373002 | 0.005194054 |
| NKX2-8 (human) | 23/256 | 0.002399456 | 0.005215484 |
| CREM (human) | 47/662 | 0.002517966 | 0.005435333 |
| REL (mouse) | 105/1752 | 0.002722846 | 0.005837334 |
| TEAD1 (human) | 94/1547 | 0.003102827 | 0.0066067 |
| ETS2 (human) | 83/1341 | 0.003383372 | 0.007146752 |
| TEAD2 (mouse) | 96/1591 | 0.003402128 | 0.007146752 |
| MZF1_5-13 (human) | 26/316 | 0.003943036 | 0.008227801 |
| JDP2 (human) | 90/1488 | 0.00423326 | 0.008774903 |
| AHR (mouse) | 34/454 | 0.004419821 | 0.009101342 |
| CREB1 (mouse) | 56/849 | 0.004570342 | 0.009349785 |
| SP4 (human) | 7/42 | 0.004812306 | 0.009727998 |
| FOXA1 (human) | 88/1457 | 0.004817379 | 0.009727998 |
| POU1F1 (mouse) | 103/1754 | 0.005223508 | 0.0104805 |
| AHR (human) | 84/1391 | 0.005828249 | 0.011548966 |
| CBEPB (human) | 145/2598 | 0.00582983 | 0.011548966 |
| MXI1 (human) | 7/46 | 0.007444466 | 0.014654829 |
| HOXD9 (human) | 91/1543 | 0.007560355 | 0.014789945 |
| PPARA (human) | 22/271 | 0.008698649 | 0.016911039 |
| FOXO3A (human) | 52/813 | 0.010418406 | 0.02012939 |
| GATA3 (human) | 138/2505 | 0.010530777 | 0.020221676 |
| CRTC3 (human) | 62/1008 | 0.011672112 | 0.022276653 |
| STAT3 (human) | 150/2759 | 0.011790928 | 0.022367034 |
| IKZF1 (mouse) | 96/1674 | 0.012251281 | 0.023100307 |
| CACYBP (mouse) | 95/1656 | 0.01256965 | 0.023558685 |
| FOS (mouse) | 97/1708 | 0.014890998 | 0.027743347 |
| NFYB (human) | 5/30 | 0.016402354 | 0.030378323 |
| BCL6 (human) | 81/1399 | 0.0166931 | 0.030734944 |
| NR5A2 (mouse) | 98/1748 | 0.019399188 | 0.035508455 |
| NR5A1 (mouse) | 61/1020 | 0.020142255 | 0.03665422 |
| CBFB (human) | 48/774 | 0.021469244 | 0.038843199 |
| JUN (mouse) | 95/1699 | 0.022386141 | 0.040269322 |
| VDR (human) | 9/88 | 0.024229416 | 0.043336042 |
| Pax6 (human) | 14/169 | 0.027667994 | 0.049205011 |
