# Supplementary material for: System analysis of the regulation of the immune response by CD147 and FOXC1 in cancer cell lines
Source: Oncotarget. 2018 Jan 11;9(16):12918–31. doi: 10.18632/oncotarget.24161 (PMC5849184; doi:10.18632/oncotarget.24161)
Supplement: Supplementary file 3 [file oncotarget-09-12918-s003.docx]

**Supplementary Table 3: The transcription factors enriched by Enrichr based on the 702 genes that negatively correlated with CD147**

| **Term** | **Overlap** | **P-value** | **Adjusted P-value** |
| --- | --- | --- | --- |
| FOXC1 (human) | 394/13142 | 4.26579E-14 | 1.22428E-11 |
| TFAP2D (human) | 68/1324 | 1.12271E-09 | 1.61108E-07 |
| TBP (human) | 104/2486 | 2.2315E-09 | 2.1348E-07 |
| TCF4 (human) | 152/4210 | 5.07261E-09 | 2.91168E-07 |
| YY1 (human) | 201/6042 | 4.59428E-09 | 2.91168E-07 |
| MYB (human) | 68/1409 | 1.21141E-08 | 4.96679E-07 |
| GATA2 (human) | 297/9985 | 1.1311E-08 | 4.96679E-07 |
| GATA6 (human) | 68/1447 | 3.24139E-08 | 1.16285E-06 |
| GATA1 (human) | 104/2679 | 9.06595E-08 | 2.89103E-06 |
| RUNX1 (human) | 136/3857 | 1.66394E-07 | 4.77551E-06 |
| USF2 (human) | 134/3792 | 1.87359E-07 | 4.88837E-06 |
| RARA (human) | 63/1379 | 2.77996E-07 | 6.64875E-06 |
| FOXL1 (human) | 185/5755 | 3.05552E-07 | 6.74565E-06 |
| MEF2A (human) | 105/2800 | 3.94203E-07 | 8.08115E-06 |
| SRF (human) | 122/3420 | 4.83523E-07 | 9.25141E-06 |
| IRF2 (human) | 63/1412 | 6.02716E-07 | 1.08112E-05 |
| NR1H3 (human) | 62/1385 | 6.70282E-07 | 1.13159E-05 |
| NR5A2 (human) | 132/3815 | 7.35863E-07 | 1.17329E-05 |
| NFKB1 (human) | 121/3497 | 2.42505E-06 | 3.6631E-05 |
| PGR (human) | 62/1447 | 2.64293E-06 | 3.79261E-05 |
| CREB1 (human) | 100/2749 | 2.88481E-06 | 3.94258E-05 |
| HNF4A (human) | 82/2127 | 3.29985E-06 | 4.30481E-05 |
| PRDM1 (human) | 62/1468 | 4.10035E-06 | 5.11652E-05 |
| SND1 (human) | 62/1473 | 4.54402E-06 | 5.43389E-05 |
| PITX2 (human) | 119/3528 | 9.67982E-06 | 0.000111124 |
| SMAD4 (mouse) | 64/1580 | 1.02004E-05 | 0.000112597 |
| FOXJ1 (human) | 61/1484 | 1.09154E-05 | 0.000116027 |
| CBEPB (human) | 93/2598 | 1.25922E-05 | 0.00012907 |
| ZFHX3 (human) | 61/1504 | 1.60408E-05 | 0.000158749 |
| NFYA (human) | 91/2549 | 1.7488E-05 | 0.000167302 |
| NFIC (human) | 140/4403 | 2.51109E-05 | 0.000232478 |
| POU2F1 (human) | 57/1399 | 2.72535E-05 | 0.000244429 |
| ETV4 (human) | 57/1403 | 2.94155E-05 | 0.000255826 |
| ETS1 (human) | 150/4827 | 3.72392E-05 | 0.000314343 |
| MAPK14 (human) | 50/1190 | 4.11827E-05 | 0.000337698 |
| ZBTB16 (human) | 61/1572 | 5.49951E-05 | 0.000438433 |
| JUN (human) | 100/2976 | 6.59186E-05 | 0.000511315 |
| NR1H2 (human) | 26/479 | 7.60792E-05 | 0.000574598 |
| LTF (human) | 54/1363 | 8.97557E-05 | 0.00066051 |
| LEF1 (human) | 143/4658 | 0.000105958 | 0.00076025 |
| POU1F1 (human) | 55/1408 | 0.000109661 | 0.000767627 |
| JUND (human) | 54/1380 | 0.000121486 | 0.000822252 |
| ATF2 (human) | 58/1517 | 0.000123195 | 0.000822252 |
| NR1I2 (human) | 54/1387 | 0.000137295 | 0.000895536 |
| NFIA (human) | 54/1416 | 0.000224735 | 0.001433312 |
| SPI1 (human) | 60/1638 | 0.00028048 | 0.001749951 |
| MYOG (human) | 53/1404 | 0.000324455 | 0.001952758 |
| RELA (human) | 91/2775 | 0.000326594 | 0.001952758 |
| HMGA1 (human) | 52/1379 | 0.000378196 | 0.002215149 |
| POU2F2 (human) | 91/2792 | 0.000396688 | 0.002276987 |
| ELF3 (human) | 55/1488 | 0.000409652 | 0.002305297 |
| SREBF1 (human) | 85/2587 | 0.000493838 | 0.002725606 |
| BCL6 (human) | 52/1399 | 0.00052016 | 0.002816716 |
| PPARG (human) | 111/3580 | 0.000530058 | 0.002817159 |
| TEAD1 (human) | 56/1547 | 0.000588338 | 0.003070055 |
| TP63 (human) | 53/1443 | 0.000599443 | 0.003072146 |
| JDP2 (human) | 54/1488 | 0.000696201 | 0.003505433 |
| SNAI1 (human) | 52/1428 | 0.000810917 | 0.003878887 |
| SNAI2 (human) | 52/1428 | 0.000810917 | 0.003878887 |
| TCF3 (human) | 52/1428 | 0.000810917 | 0.003878887 |
| IRF8 (human) | 55/1550 | 0.0010225 | 0.004810779 |
| ELK4 (human) | 52/1453 | 0.001169501 | 0.005413658 |
| CRTC3 (human) | 39/1008 | 0.001284648 | 0.005775182 |
| NR5A1 (human) | 50/1389 | 0.001287845 | 0.005775182 |
| SMARCA2 (human) | 54/1536 | 0.001381392 | 0.006099379 |
| ESR1 (human) | 53/1513 | 0.001644849 | 0.0071526 |
| PITX1 (human) | 51/1448 | 0.001797481 | 0.007699657 |
| FOXF2 (human) | 49/1379 | 0.001853049 | 0.007820956 |
| XBP1 (human) | 26/605 | 0.002119917 | 0.00867846 |
| FOXF1 (human) | 48/1352 | 0.002089143 | 0.00867846 |
| GFI1 (human) | 50/1427 | 0.002199756 | 0.00867846 |
| RBPJ (human) | 53/1535 | 0.002207413 | 0.00867846 |
| E2F1 (human) | 123/4207 | 0.00216777 | 0.00867846 |
| HOXA5 (human) | 92/3000 | 0.002305268 | 0.008940702 |
| RELB (human) | 51/1469 | 0.002389707 | 0.009133595 |
| SMAD4 (human) | 53/1542 | 0.002418652 | 0.009133595 |
| NFKB1 (mouse) | 99/3284 | 0.002590104 | 0.009654023 |
| CEBPB (human) | 49/1420 | 0.003238298 | 0.011915277 |
| RXRA (human) | 49/1422 | 0.003324297 | 0.012076877 |
| SP1 (mouse) | 74/2360 | 0.003720141 | 0.013346005 |
| HIF1A (human) | 53/1586 | 0.004195427 | 0.014865277 |
| NFAT2 (human) | 47/1376 | 0.004670873 | 0.016087234 |
| HNF1A (human) | 60/1855 | 0.004708459 | 0.016087234 |
| WT1 (human) | 82/2689 | 0.004622712 | 0.016087234 |
| RUNX2 (human) | 49/1458 | 0.005245841 | 0.017597823 |
| CEBPD (human) | 50/1495 | 0.005273215 | 0.017597823 |
| RORB (human) | 37/1035 | 0.005867677 | 0.018921609 |
| ZNF354C (human) | 48/1430 | 0.005823897 | 0.018921609 |
| NRF1 (human) | 46/1356 | 0.00574291 | 0.018921609 |
| LEF1 (mouse) | 56/1730 | 0.006146989 | 0.019602064 |
| GATA3 (human) | 76/2505 | 0.00714077 | 0.022520891 |
| ETS2 (human) | 45/1341 | 0.007494293 | 0.023378937 |
| STAT5B (human) | 46/1379 | 0.007624427 | 0.023529145 |
| SP3 (mouse) | 39/1129 | 0.008035787 | 0.024534798 |
| STAT3 (human) | 82/2759 | 0.008574236 | 0.025903218 |
| FOS (human) | 46/1394 | 0.009112045 | 0.027232805 |
| HOXD9 (human) | 50/1543 | 0.009204119 | 0.027232805 |
| NR2F1 (human) | 37/1073 | 0.009922666 | 0.029059237 |
| NR3C1 (human) | 51/1590 | 0.010234808 | 0.029670604 |
| STAT1 (human) | 53/1667 | 0.01045589 | 0.030008406 |
| IKZF1 (human) | 45/1373 | 0.010960172 | 0.031000412 |
| MYOD1 (mouse) | 53/1672 | 0.011017568 | 0.031000412 |
| NRF1 (mouse) | 53/1676 | 0.011484819 | 0.03200139 |
| MIR133B (human) | 48/1500 | 0.013006769 | 0.035893679 |
| EGR1 (mouse) | 51/1617 | 0.013602915 | 0.037181301 |
| CRX (human) | 44/1362 | 0.014717157 | 0.039847396 |
| MAX (human) | 59/1934 | 0.015351136 | 0.041175476 |
| Myb (mouse) | 58/1902 | 0.016287003 | 0.043281202 |
| TFAP2C (human) | 47/1485 | 0.016510212 | 0.043471841 |
| NKX2-8 (human) | 12/256 | 0.017457405 | 0.045547957 |
| VDR (human) | 6/88 | 0.018508517 | 0.047855354 |
