# Supplementary material for: System analysis of the regulation of the immune response by CD147 and FOXC1 in cancer cell lines
Source: Oncotarget. 2018 Jan 11;9(16):12918–31. doi: 10.18632/oncotarget.24161 (PMC5849184; doi:10.18632/oncotarget.24161)
Supplement: Supplementary file 4 [file oncotarget-09-12918-s004.docx]

**Supplementary Table 4: The transcription factors enriched by Enrichr based on the Immu_Genes from InnateDB and IRIS databases**

| **Term** | **Overlap** | **P-value** | **Adjusted P-value** |
| --- | --- | --- | --- |
| NR5A2 (human) | 180/3815 | 1.59322E-22 | 5.1461E-20 |
| TCF4 (human) | 172/4210 | 2.12094E-15 | 3.42532E-13 |
| CBEPB (human) | 117/2598 | 6.07509E-13 | 5.78364E-11 |
| PPARG (human) | 146/3580 | 7.1624E-13 | 5.78364E-11 |
| NFKB1 (human) | 142/3497 | 2.26078E-12 | 1.46046E-10 |
| LEF1 (human) | 173/4658 | 6.33182E-12 | 3.40863E-10 |
| STAT3 (human) | 115/2759 | 1.08865E-10 | 5.02336E-09 |
| ELF3 (human) | 75/1488 | 1.45178E-10 | 5.21029E-09 |
| NFKB1 (mouse) | 130/3284 | 1.32552E-10 | 5.21029E-09 |
| TEAD1 (human) | 76/1547 | 3.28987E-10 | 1.06263E-08 |
| SND1 (human) | 73/1473 | 5.32252E-10 | 1.56288E-08 |
| USF2 (human) | 141/3792 | 1.10895E-09 | 2.98491E-08 |
| ETS2 (human) | 67/1341 | 2.14687E-09 | 4.84395E-08 |
| NR3C1 (human) | 75/1590 | 2.39451E-09 | 4.84395E-08 |
| RELA (human) | 111/2775 | 2.39948E-09 | 4.84395E-08 |
| JUN (human) | 117/2976 | 2.10835E-09 | 4.84395E-08 |
| CREB1 (human) | 109/2749 | 5.4845E-09 | 1.04205E-07 |
| ETS1 (human) | 166/4827 | 6.15108E-09 | 1.10378E-07 |
| TFAP2A (mouse) | 76/1663 | 6.83797E-09 | 1.16246E-07 |
| PGR (human) | 69/1447 | 7.38183E-09 | 1.19217E-07 |
| RUNX2 (human) | 69/1458 | 9.84633E-09 | 1.51446E-07 |
| TBP (human) | 100/2486 | 1.33869E-08 | 1.96543E-07 |
| FOS (human) | 66/1394 | 2.09046E-08 | 2.93573E-07 |
| GATA2 (human) | 290/9985 | 3.43616E-08 | 4.6245E-07 |
| SREBF2 (human) | 64/1361 | 4.42273E-08 | 5.54788E-07 |
| IRF8 (human) | 70/1550 | 4.46579E-08 | 5.54788E-07 |
| MIR133B (human) | 68/1500 | 6.17748E-08 | 7.39009E-07 |
| GATA3 (human) | 98/2505 | 7.04772E-08 | 8.13005E-07 |
| NFE2 (human) | 102/2681 | 1.3473E-07 | 1.50062E-06 |
| RUNX1 (human) | 134/3857 | 1.73148E-07 | 1.86423E-06 |
| POU2F2 (human) | 104/2792 | 2.66313E-07 | 2.77481E-06 |
| SPI1 (human) | 70/1638 | 3.29934E-07 | 3.33027E-06 |
| FOXC1 (human) | 356/13142 | 4.59796E-07 | 4.50043E-06 |
| NFAT2 (human) | 61/1376 | 6.36351E-07 | 6.04533E-06 |
| TP63 (human) | 63/1443 | 6.7642E-07 | 6.24239E-06 |
| BCL6 (human) | 61/1399 | 1.07189E-06 | 9.61722E-06 |
| ETV4 (human) | 61/1403 | 1.1717E-06 | 1.02287E-05 |
| NR5A1 (human) | 60/1389 | 1.75808E-06 | 1.45605E-05 |
| KLF13 (human) | 62/1453 | 1.7157E-06 | 1.45605E-05 |
| GATA1 (human) | 97/2679 | 2.39929E-06 | 1.93743E-05 |
| TP53 (human) | 102/2866 | 2.63144E-06 | 2.07306E-05 |
| MYB (human) | 60/1409 | 2.70908E-06 | 2.08341E-05 |
| HMGA1 (human) | 59/1379 | 2.86702E-06 | 2.1536E-05 |
| MIB2 (human) | 62/1490 | 3.71854E-06 | 2.72975E-05 |
| PRDM1 (human) | 61/1468 | 4.66377E-06 | 3.34755E-05 |
| NFATC2 (human) | 13/117 | 5.79025E-06 | 3.97926E-05 |
| SPI1 (mouse) | 67/1680 | 5.75918E-06 | 3.97926E-05 |
| ATF2 (human) | 62/1517 | 6.38683E-06 | 4.2978E-05 |
| PITX2 (human) | 118/3528 | 6.85772E-06 | 4.5205E-05 |
| RBPJ (human) | 62/1535 | 9.06293E-06 | 5.85465E-05 |
| RELB (human) | 60/1469 | 9.25091E-06 | 5.85891E-05 |
| NFIA (human) | 58/1416 | 1.21304E-05 | 7.53484E-05 |
| SNAI1 (human) | 58/1428 | 1.53413E-05 | 9.0095E-05 |
| SNAI2 (human) | 58/1428 | 1.53413E-05 | 9.0095E-05 |
| TCF3 (human) | 58/1428 | 1.53413E-05 | 9.0095E-05 |
| FOXL1 (human) | 173/5755 | 1.67656E-05 | 9.67017E-05 |
| YY1 (human) | 180/6042 | 1.75455E-05 | 9.94244E-05 |
| POU1F1 (human) | 57/1408 | 1.99242E-05 | 0.000110853 |
| RUNX2 (mouse) | 67/1750 | 2.02487E-05 | 0.000110853 |
| STAT5B (human) | 56/1379 | 2.17386E-05 | 0.000117026 |
| LTF (human) | 55/1363 | 3.04669E-05 | 0.000161325 |
| GATA6 (human) | 57/1447 | 4.13714E-05 | 0.000215532 |
| TFAP2C (human) | 58/1485 | 4.43831E-05 | 0.000227552 |
| POU2F1 (human) | 55/1399 | 5.95702E-05 | 0.000300643 |
| HOXD9 (human) | 59/1543 | 6.70827E-05 | 0.000333349 |
| CRTC3 (human) | 43/1008 | 7.05811E-05 | 0.000340264 |
| ELK1 (human) | 58/1511 | 7.00697E-05 | 0.000340264 |
| MZF1_1-4 (human) | 91/2705 | 7.64348E-05 | 0.000363065 |
| CEBPD (human) | 57/1495 | 9.62507E-05 | 0.000450565 |
| ZFHX3 (human) | 57/1504 | 0.000112036 | 0.000516965 |
| SMAD4 (human) | 58/1542 | 0.000118178 | 0.000537628 |
| JUND (human) | 53/1380 | 0.000142898 | 0.000641057 |
| CRTC1 (human) | 44/1086 | 0.000176003 | 0.000778752 |
| PRDM1 (mouse) | 14/196 | 0.000232604 | 0.001015285 |
| HNF4A (human) | 73/2127 | 0.000239069 | 0.001029591 |
| IRF2 (human) | 53/1412 | 0.000244784 | 0.001040331 |
| SAMD9L (human) | 53/1415 | 0.000257101 | 0.001078487 |
| SRF (human) | 107/3420 | 0.000263278 | 0.001090239 |
| FOS (mouse) | 61/1708 | 0.000300029 | 0.001226701 |
| HNF1A (human) | 65/1855 | 0.000312682 | 0.001262455 |
| MAPK14 (human) | 46/1190 | 0.000342938 | 0.001367518 |
| ETV4 (mouse) | 62/1773 | 0.000453846 | 0.001787709 |
| JDP2 (human) | 54/1488 | 0.000466504 | 0.001815431 |
| PURA (human) | 24/492 | 0.000486493 | 0.001870681 |
| IKZF1 (mouse) | 59/1674 | 0.000518155 | 0.00196899 |
| MYOG (human) | 51/1404 | 0.000655461 | 0.002452657 |
| JUND (mouse) | 61/1764 | 0.000660623 | 0.002452657 |
| CACYBP (mouse) | 58/1656 | 0.000668674 | 0.002454339 |
| IRF8 (mouse) | 61/1770 | 0.000716325 | 0.002599698 |
| NR3C1 (mouse) | 61/1775 | 0.000765916 | 0.002748789 |
| NR1H2 (human) | 23/479 | 0.000774554 | 0.002749241 |
| CACYBP (human) | 49/1350 | 0.000847124 | 0.002974141 |
| FOXF1 (human) | 49/1352 | 0.000873246 | 0.003032886 |
| MIR138 (human) | 49/1356 | 0.000927644 | 0.003121137 |
| REL (mouse) | 60/1752 | 0.000917129 | 0.003121137 |
| Gata1 (mouse) | 63/1863 | 0.000927012 | 0.003121137 |
| Stat3 (mouse) | 60/1762 | 0.001045795 | 0.003482391 |
| JUN (mouse) | 58/1699 | 0.001197061 | 0.003945417 |
| STAT1 (mouse) | 7/68 | 0.001264602 | 0.004125923 |
| SPIB (human) | 31/760 | 0.001402786 | 0.004516669 |
| MYOG (mouse) | 57/1675 | 0.001412333 | 0.004516669 |
| MEF2A (human) | 86/2800 | 0.00190665 | 0.006037725 |
| RORB (mouse) | 38/1020 | 0.00205917 | 0.006334399 |
| HIF1A (mouse) | 41/1124 | 0.002020505 | 0.006334399 |
| STAT1 (human) | 56/1667 | 0.002041955 | 0.006334399 |
| NR2F1 (mouse) | 38/1021 | 0.002092764 | 0.006377008 |
| CEBPB (human) | 49/1420 | 0.002310917 | 0.006975945 |
| NR4A2 (human) | 7/79 | 0.002788538 | 0.008339793 |
| RELA (mouse) | 55/1656 | 0.002819971 | 0.008356428 |
| USF1 (human) | 49/1441 | 0.003052246 | 0.008962505 |
| APEX1 (human) | 47/1373 | 0.00326434 | 0.009414124 |
| IKZF1 (human) | 47/1373 | 0.00326434 | 0.009414124 |
| MYC (human) | 45/1302 | 0.003343389 | 0.009556766 |
| CBFB (human) | 30/774 | 0.003418537 | 0.009685856 |
| FOXO3A (human) | 31/813 | 0.003714382 | 0.010432568 |
| RXRA (human) | 48/1422 | 0.003834359 | 0.010676707 |
| HINFP (human) | 90/3047 | 0.004377956 | 0.012086152 |
| HIVEP1 (human) | 12/218 | 0.004942254 | 0.013528374 |
| SND1 (mouse) | 7/90 | 0.005435088 | 0.014752381 |
| FOXA1 (human) | 48/1457 | 0.005915048 | 0.015921339 |
| NFYA (human) | 76/2549 | 0.007069553 | 0.018871617 |
| TFAP2D (human) | 44/1324 | 0.007139419 | 0.018901905 |
| MAX (human) | 60/1934 | 0.007586331 | 0.019921828 |
| REL (human) | 16/353 | 0.007715268 | 0.019936254 |
| USF1 (mouse) | 41/1219 | 0.007667598 | 0.019936254 |
| KLF4 (human) | 48/1485 | 0.008208648 | 0.021042804 |
| FOXF2 (human) | 45/1379 | 0.008842162 | 0.022488332 |
| UBTF (human) | 48/1493 | 0.008987175 | 0.022678575 |
| NR1H3 (human) | 45/1385 | 0.009483049 | 0.023744378 |
| NR1I2 (human) | 45/1387 | 0.009705106 | 0.024113457 |
| KLF11 (human) | 45/1388 | 0.009817751 | 0.024207125 |
| MZF1 (human) | 45/1389 | 0.009931482 | 0.02424025 |
| GFI1 (human) | 46/1427 | 0.009981279 | 0.02424025 |
| POU3F1 (human) | 9/162 | 0.013206339 | 0.031833191 |
| Nkx3-2 (mouse) | 37/1122 | 0.014428251 | 0.034520927 |
| ARID3A (mouse) | 57/1893 | 0.015688946 | 0.037261246 |
| ZNF148 (human) | 49/1591 | 0.016943726 | 0.039947617 |
| FOXO3A (mouse) | 22/597 | 0.018492697 | 0.043283631 |
| LEF1 (mouse) | 52/1730 | 0.021069264 | 0.048959513 |
