# Supplementary material for: System analysis of the regulation of the immune response by CD147 and FOXC1 in cancer cell lines
Source: Oncotarget. 2018 Jan 11;9(16):12918–31. doi: 10.18632/oncotarget.24161 (PMC5849184; doi:10.18632/oncotarget.24161)
Supplement: Supplementary file 5 [file oncotarget-09-12918-s005.doc]

**Supplementary Table 7: The GO biology processes enriched with genes that negatively correlate with CD147 in cancer cell lines**

| **Term** | **Overlap** | **P-value** | **Adjusted P-value** |
| --- | --- | --- | --- |
| Antigen receptor-mediated signaling pathway (GO:0050851) | 28/127 | 6.50896E-15 | 1.76783E-11 |
| B cell receptor signaling pathway (GO:0050853) | 17/33 | 2.16396E-14 | 2.93865E-11 |
| Lymphocyte activation (GO:0046649) | 40/304 | 5.74936E-14 | 5.20509E-11 |
| Leukocyte activation (GO:0045321) | 43/373 | 3.85456E-13 | 2.61725E-10 |
| Immune response-activating signal transduction (GO:0002757) | 45/440 | 4.7019E-12 | 2.55407E-09 |
| Activation of immune response (GO:0002253) | 46/487 | 3.26698E-11 | 1.47885E-08 |
| Immune response-activating cell surface receptor signaling pathway (GO:0002429) | 36/324 | 9.03113E-11 | 3.50408E-08 |
| Regulation of cell activation (GO:0050865) | 39/420 | 1.77439E-09 | 6.02404E-07 |
| Regulation of lymphocyte activation (GO:0051249) | 34/344 | 4.89699E-09 | 1.4778E-06 |
| Immune response-regulating cell surface receptor signaling pathway (GO:0002768) | 39/444 | 7.44682E-09 | 2.02256E-06 |
| Regulation of leukocyte activation (GO:0002694) | 36/390 | 8.9117E-09 | 2.20038E-06 |
| B cell activation (GO:0042113) | 19/128 | 4.96581E-08 | 1.12393E-05 |
| T cell activation (GO:0042110) | 23/198 | 1.19225E-07 | 2.49088E-05 |
| Regulation of B cell activation (GO:0050864) | 15/92 | 4.44148E-07 | 8.61647E-05 |
| Chromatin modification (GO:0016568) | 36/475 | 8.35806E-07 | 0.000151337 |
| Regulation of B cell proliferation (GO:0030888) | 11/54 | 2.34248E-06 | 0.000397636 |
| Regulation of T cell activation (GO:0050863) | 24/259 | 2.68394E-06 | 0.000428798 |
| T cell receptor signaling pathway (GO:0050852) | 14/99 | 4.81838E-06 | 0.000727039 |
| Positive regulation of cell activation (GO:0050867) | 24/272 | 5.83151E-06 | 0.000833599 |
| Positive regulation of gtpase activity (GO:0043547) | 34/482 | 7.35085E-06 | 0.000990781 |
| Positive regulation of GTP catabolic process (GO:0033126) | 34/483 | 7.66068E-06 | 0.000990781 |
| Positive regulation of lymphocyte activation (GO:0051251) | 22/243 | 9.83463E-06 | 0.001163205 |
| Positive regulation of leukocyte activation (GO:0002696) | 23/262 | 9.85041E-06 | 0.001163205 |
| Lymphocyte proliferation (GO:0046651) | 12/81 | 1.50873E-05 | 0.00170608 |
| Leukocyte differentiation (GO:0002521) | 22/251 | 1.5704E-05 | 0.00170608 |
| Mononuclear cell proliferation (GO:0032943) | 12/83 | 1.87883E-05 | 0.00196265 |
| Positive regulation of B cell activation (GO:0050871) | 10/58 | 2.44581E-05 | 0.002460307 |
| Leukocyte proliferation (GO:0070661) | 12/89 | 3.5036E-05 | 0.003398496 |
| Regulation of lymphocyte differentiation (GO:0045619) | 14/124 | 4.79553E-05 | 0.004491261 |
| Histone modification (GO:0016570) | 23/293 | 5.07611E-05 | 0.004595569 |
| Covalent chromatin modification (GO:0016569) | 23/296 | 5.86963E-05 | 0.005142555 |
| T cell proliferation (GO:0042098) | 8/41 | 7.43999E-05 | 0.00631469 |
| Hemostasis (GO:0007599) | 31/478 | 8.30261E-05 | 0.006833302 |
| Regulation of lymphocyte proliferation (GO:0050670) | 16/170 | 0.000105135 | 0.008398418 |
| Regulation of mononuclear cell proliferation (GO:0032944) | 16/171 | 0.000111979 | 0.008689559 |
| Lymphocyte differentiation (GO:0030098) | 16/172 | 0.000119209 | 0.008993686 |
| B cell homeostasis (GO:0001782) | 6/23 | 0.000154709 | 0.01050474 |
| Regulation of leukocyte proliferation (GO:0070663) | 16/176 | 0.000152372 | 0.01050474 |
| Blood coagulation (GO:0007596) | 30/472 | 0.000150848 | 0.01050474 |
| Coagulation (GO:0050817) | 30/472 | 0.000150848 | 0.01050474 |
| Positive regulation of lymphocyte differentiation (GO:0045621) | 10/75 | 0.000169856 | 0.011251916 |
| Adaptive immune response (GO:0002250) | 11/91 | 0.000178383 | 0.011535435 |
| Positive regulation of B cell proliferation (GO:0030890) | 7/36 | 0.000214407 | 0.013542518 |
| Small gtpase mediated signal transduction (GO:0007264) | 28/439 | 0.00023669 | 0.014610215 |
| Regulation of adaptive immune response (GO:0002819) | 12/112 | 0.000257168 | 0.015521528 |
| Positive regulation of T cell activation (GO:0050870) | 16/189 | 0.000321786 | 0.01868542 |
| Activation of innate immune response (GO:0002218) | 14/151 | 0.000323349 | 0.01868542 |
| Regulation of leukocyte differentiation (GO:1902105) | 17/210 | 0.000343391 | 0.019430193 |
| Mast cell activation (GO:0045576) | 5/17 | 0.00035071 | 0.019439372 |
| Negative regulation of cell activation (GO:0050866) | 13/135 | 0.000375455 | 0.02039472 |
| Positive regulation of leukocyte differentiation (GO:1902107) | 12/118 | 0.000398259 | 0.020801384 |
| Regulation of innate immune response (GO:0045088) | 19/254 | 0.000397309 | 0.020801384 |
| Negative regulation of lymphocyte activation (GO:0051250) | 11/102 | 0.00043808 | 0.022449527 |
| Negative regulation of leukocyte activation (GO:0002695) | 12/120 | 0.00045787 | 0.022794792 |
| Myeloid leukocyte activation (GO:0002274) | 10/86 | 0.000461603 | 0.022794792 |
| Regulation of adaptive immune response based on somatic recombination of immune receptors built from immunoglobulin superfamily domains (GO:0002822) | 11/103 | 0.00047251 | 0.022916755 |
| Regulation of germinal center formation (GO:0002634) | 4/10 | 0.000553936 | 0.025939497 |
| Centriole replication (GO:0007099) | 4/10 | 0.000553936 | 0.025939497 |
| Pattern recognition receptor signaling pathway (GO:0002221) | 13/142 | 0.000585053 | 0.026932258 |
| Innate immune response-activating signal transduction (GO:0002758) | 13/144 | 0.000660548 | 0.029900823 |
| Positive regulation of B cell differentiation (GO:0045579) | 4/11 | 0.000738168 | 0.032732245 |
| Leukocyte migration (GO:0050900) | 17/226 | 0.000747201 | 0.032732245 |
| T cell costimulation (GO:0031295) | 9/76 | 0.000781728 | 0.033701184 |
| Regulation of B cell differentiation (GO:0045577) | 5/21 | 0.00079625 | 0.033790856 |
| Lymphocyte costimulation (GO:0031294) | 9/77 | 0.000851204 | 0.035567236 |
| Leukocyte activation involved in immune response (GO:0002366) | 11/112 | 0.000897313 | 0.036374669 |
| Cell activation involved in immune response (GO:0002263) | 11/112 | 0.000897313 | 0.036374669 |
| Positive regulation of innate immune response (GO:0045089) | 15/190 | 0.000957314 | 0.038236264 |
| Regulation of antigen receptor-mediated signaling pathway (GO:0050854) | 6/35 | 0.001091893 | 0.040190014 |
| B cell differentiation (GO:0030183) | 9/80 | 0.001090297 | 0.040190014 |
| T cell differentiation (GO:0030217) | 10/97 | 0.001084893 | 0.040190014 |
| Regulation of T cell differentiation (GO:0045580) | 10/97 | 0.001084893 | 0.040190014 |
| Positive regulation of lymphocyte proliferation (GO:0050671) | 11/115 | 0.001095015 | 0.040190014 |
| Regulation of small gtpase mediated signal transduction (GO:0051056) | 18/256 | 0.001093747 | 0.040190014 |
| Mrna splice site selection (GO:0006376) | 5/23 | 0.0011343 | 0.041076799 |
| Positive regulation of mononuclear cell proliferation (GO:0032946) | 11/116 | 0.001168394 | 0.041754718 |
| Transcription from RNA polymerase II promoter (GO:0006366) | 27/470 | 0.001358358 | 0.047913003 |
| Positive regulation of leukocyte proliferation (GO:0070665) | 11/119 | 0.001413201 | 0.049208397 |
| Positive regulation of interferon-gamma production (GO:0032729) | 7/52 | 0.001525149 | 0.051778811 |
| Regulation of immune effector process (GO:0002697) | 18/264 | 0.001511729 | 0.051778811 |
| Centriole assembly (GO:0098534) | 4/14 | 0.001544522 | 0.051789173 |
| Negative regulation of immune system process (GO:0002683) | 20/311 | 0.001640417 | 0.054333816 |
| Toll-like receptor signaling pathway (GO:0002224) | 11/122 | 0.001698576 | 0.055582324 |
| Positive regulation of Ras gtpase activity (GO:0032320) | 19/292 | 0.001874718 | 0.06061587 |
| Granulocyte differentiation (GO:0030851) | 4/15 | 0.001912025 | 0.061094821 |
| Regulation of leukocyte apoptotic process (GO:2000106) | 8/71 | 0.002021523 | 0.063842505 |
| Positive regulation of defense response (GO:0031349) | 18/272 | 0.002057824 | 0.064241949 |
| Dentate gyrus development (GO:0021542) | 4/16 | 0.002335881 | 0.072093788 |
| Adaptive immune response based on somatic recombination of immune receptors built from immunoglobulin superfamily domains (GO:0002460) | 7/57 | 0.002454176 | 0.074893744 |
| Regulation of Ras gtpase activity (GO:0032318) | 21/349 | 0.002704873 | 0.081627071 |
| Negative regulation of B cell activation (GO:0050869) | 5/29 | 0.002784939 | 0.083119727 |
| Lymph node development (GO:0048535) | 4/17 | 0.002820191 | 0.083256949 |
| Lymphocyte homeostasis (GO:0002260) | 6/44 | 0.003100395 | 0.090544873 |
| Regulation of defense response to virus (GO:0050688) | 8/77 | 0.00320922 | 0.092725969 |
| Myeloid cell differentiation (GO:0030099) | 12/154 | 0.00331352 | 0.094731795 |
